# Supplementary material for: Hydration free energy is an incomplete predictor of globular protein incorporation into condensates
Source: Biophys J. 2025 Dec 4;125(16):4147–56. doi: 10.1016/j.bpj.2025.12.005 (PMC12798646; doi:10.1016/j.bpj.2025.12.005)
Supplement: Supplementary file 1 [file mmc1.pdf]

Biophysical Journal, Volume *125*

Supplemental information

Hydration free energy is an incomplete predictor of globular protein incorporation into condensates

Sophie Anderson, Malcolm Harrison, and Gregory L. Dignon

Supporting Information: Hydration free energy is an incomplete predictor of globular protein incorporation into condensates

Sophie Anderson[1,2], Malcolm Harrison[3], and Gregory L Dignon[3,*]

[1]Department of Biochemistry, Vassar College, Poughkeepsie, NY 12604, United States
[2]Current Address: Department of Epidemiology Columbia University, New York, NY 10032, United States.
[3]Department of Chemical and Biochemical Engineering, Rutgers University, Piscataway, NJ 08854, United States
[*]Correspondence: gregory.dignon@rutgers.edu

1. Supplementary Figures

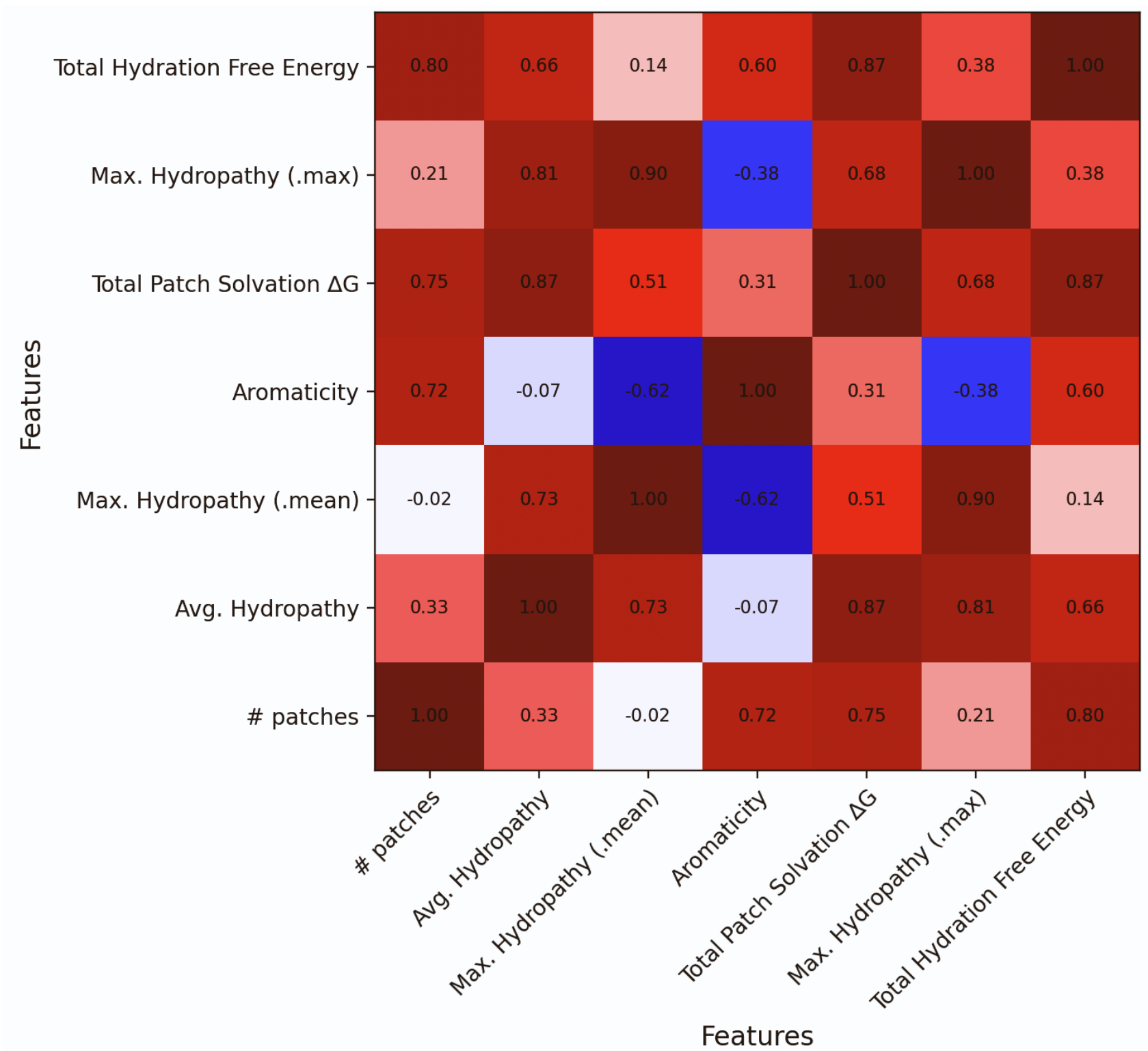

Fig S1: Analysis of different outputs from hi-patch software[3] (See Table S1) and their relatedness when applied to the 17 variants of GFP. Covariance will be 1 if parameters are perfectly correlated, and -1 if perfectly anticorrelated. A value close to 0 indicates they are uncorrelated.

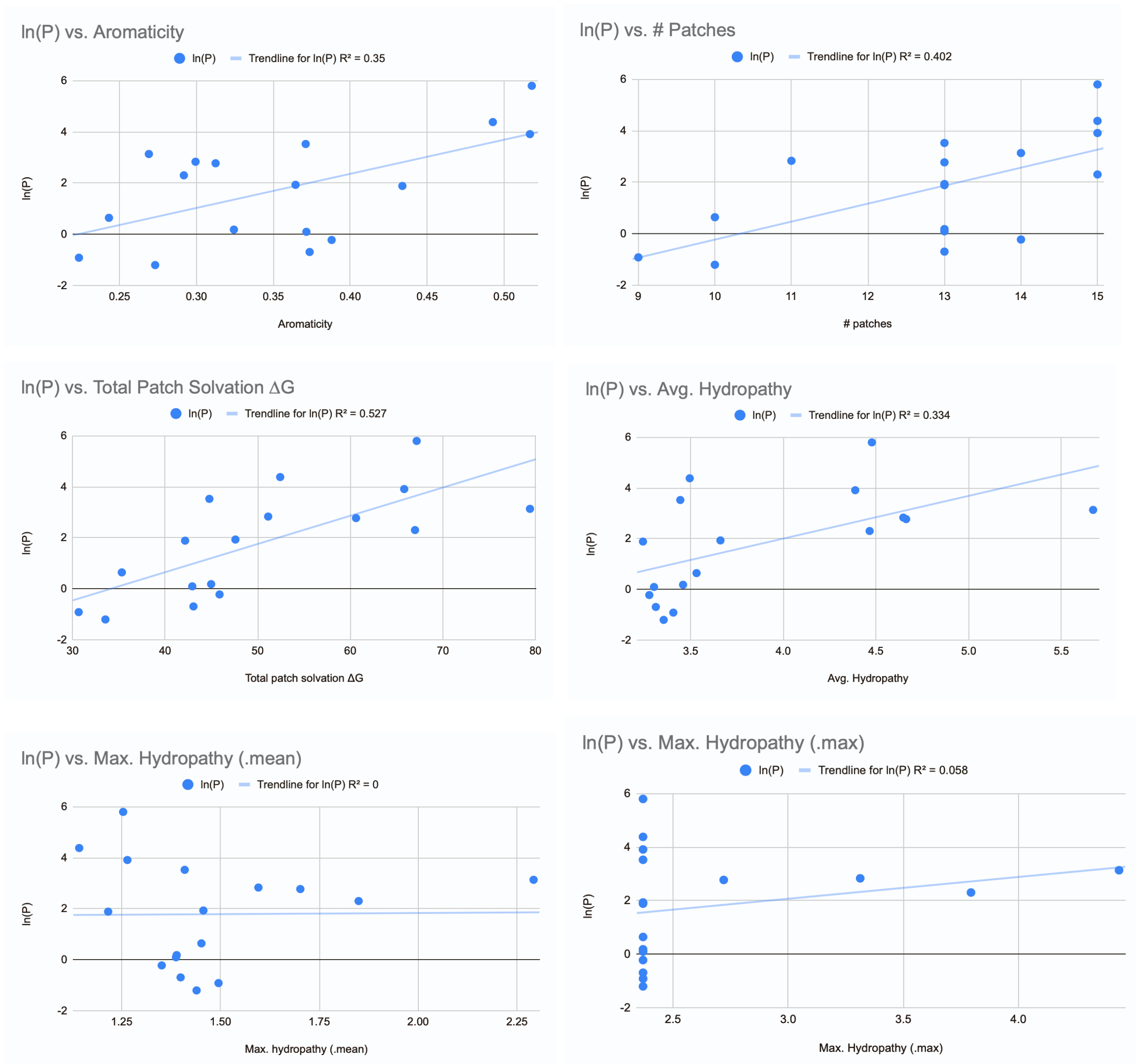

Fig. S2: predictions of ln(P) from other hi-patch outputs are not as robust as with the full hydration free energy calculation.

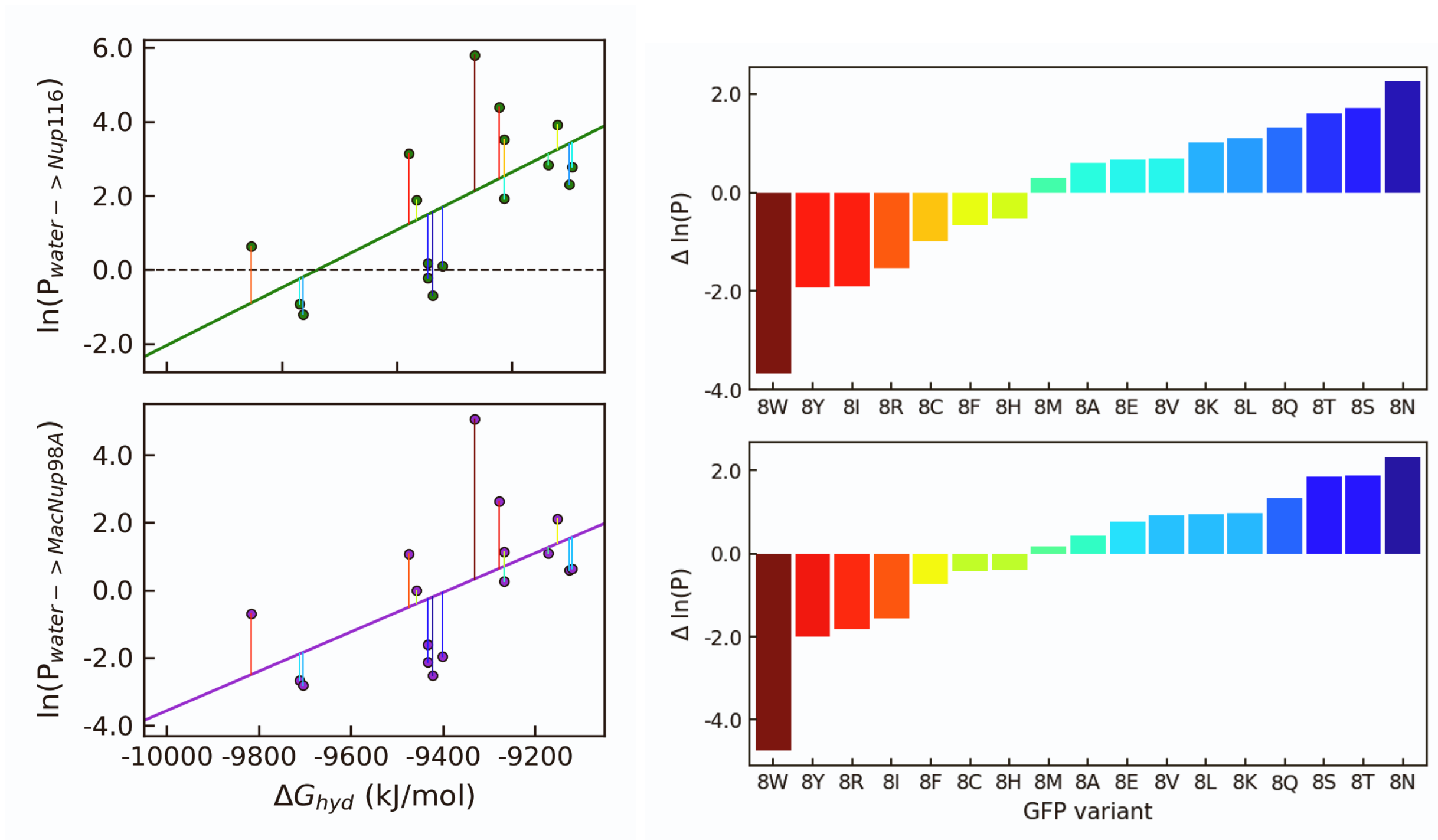

Fig. S3: Predictive model and residuals for static calculation of hydration free energy using the eef1 force field implemented in CHARMM[4].

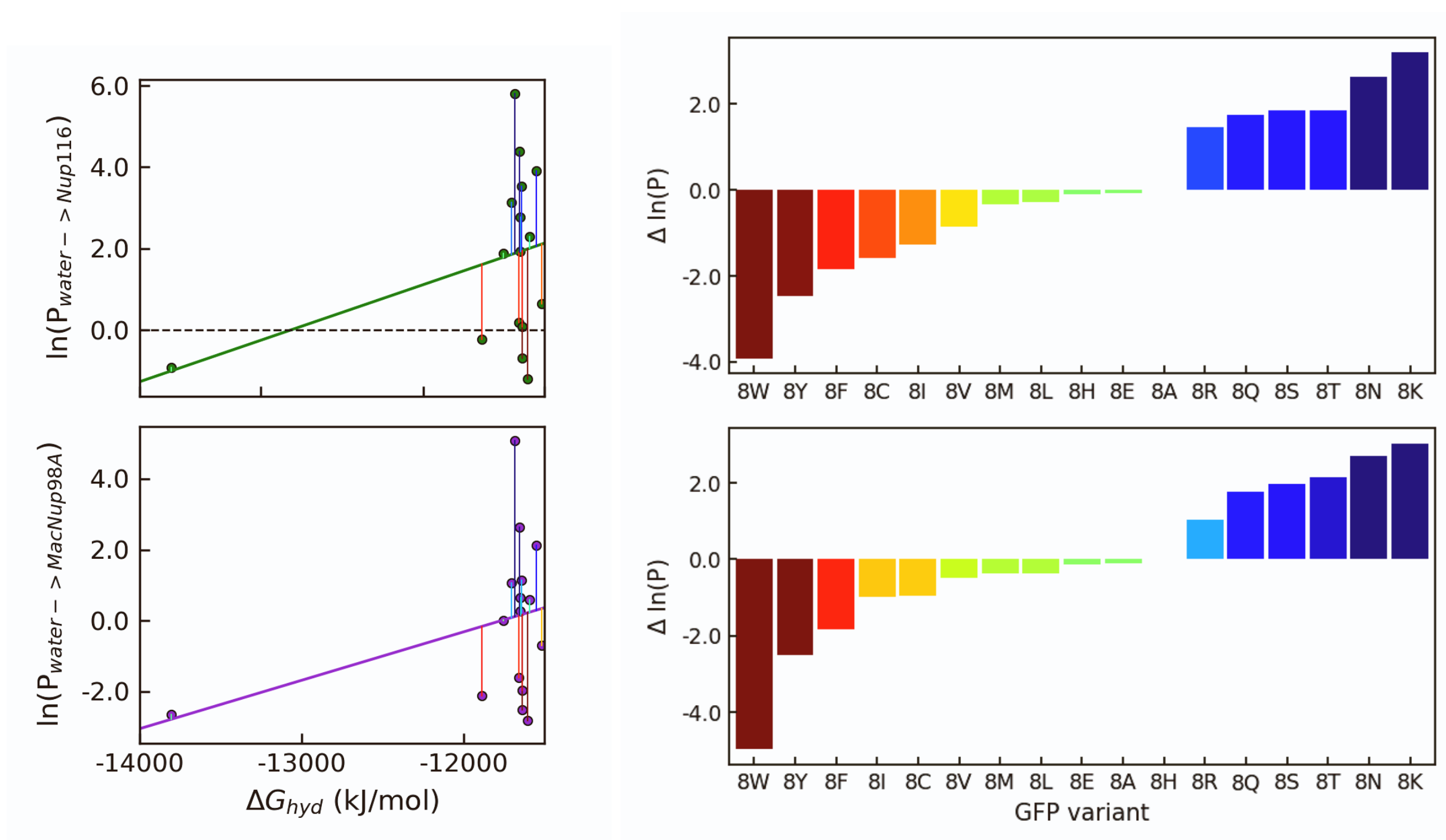

Fig S4: Predictive model and residuals for static calculation of hydration free energy using the adaptive Poisson-Boltzmann solver (APBS)[5].

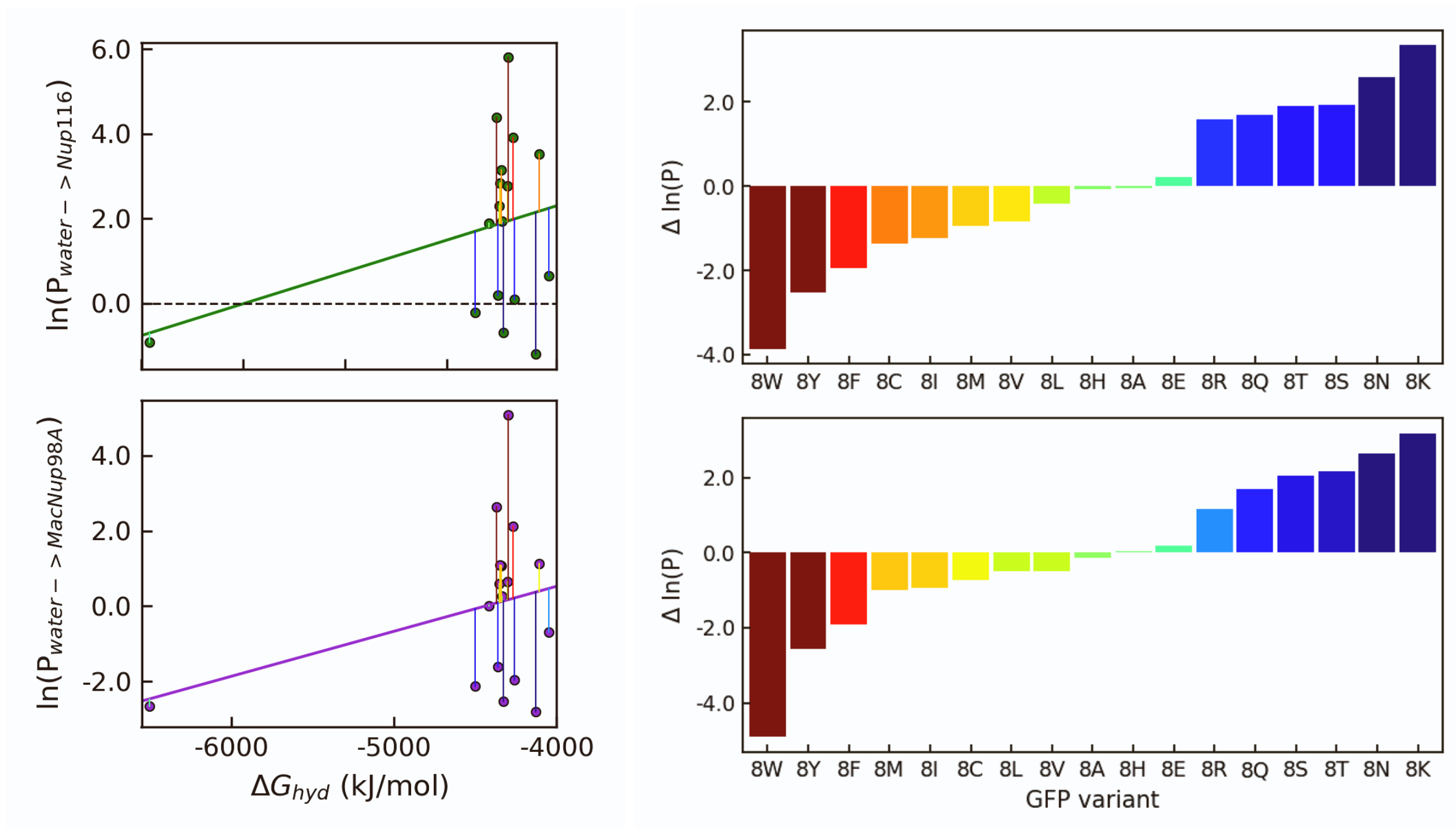

Fig. S5: Predictive model and residuals for dynamic hydration free energy calculations using MD simulation with GBNeck implicit solvent[6].

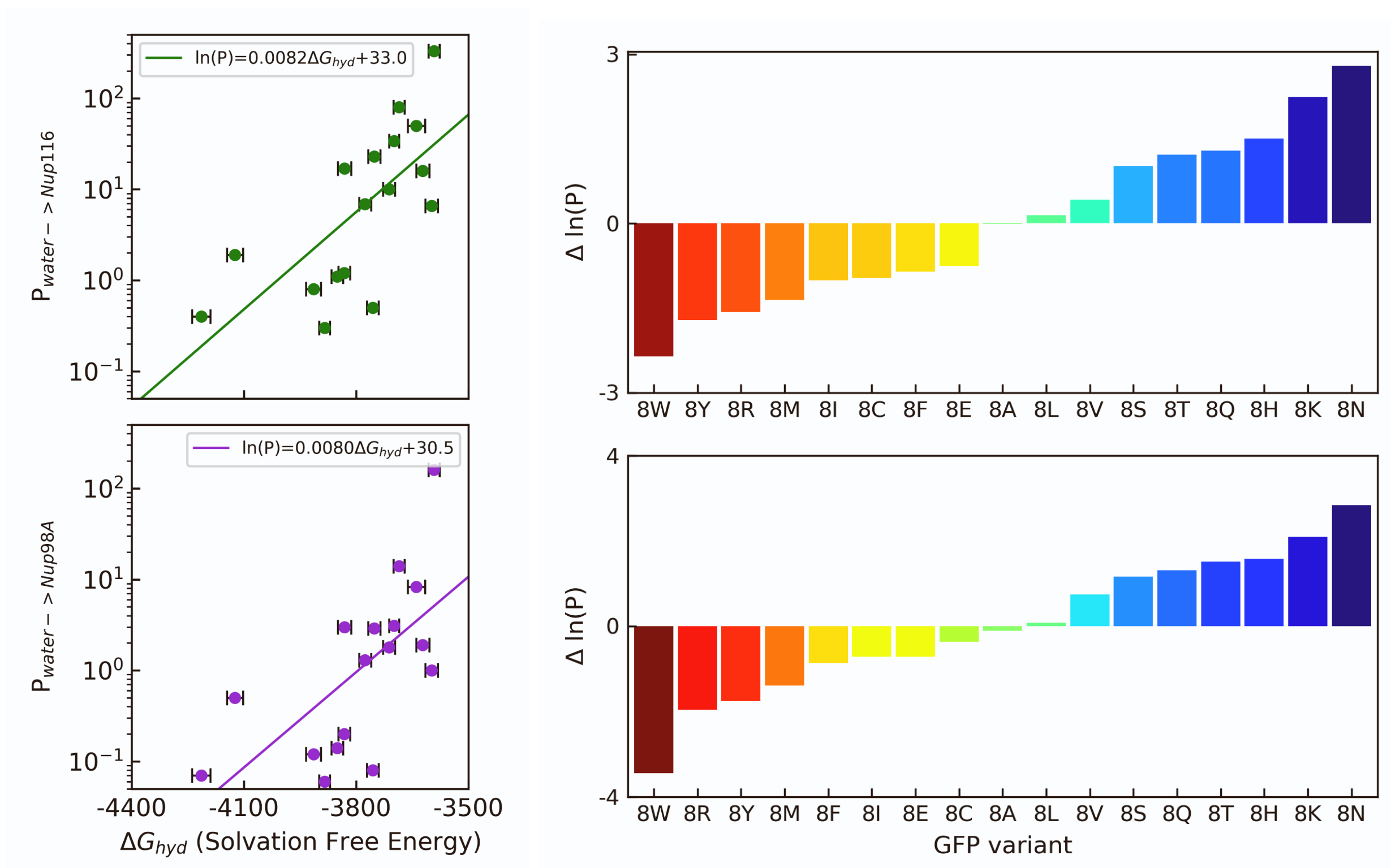

Fig. S6: Predictive model and residuals for dynamic explicit solvent simulation trajectory analyzed using hi-patch to calculate hydration free energy. Error bars are SEM from the simulation calculated by block averaging with 5 blocks.

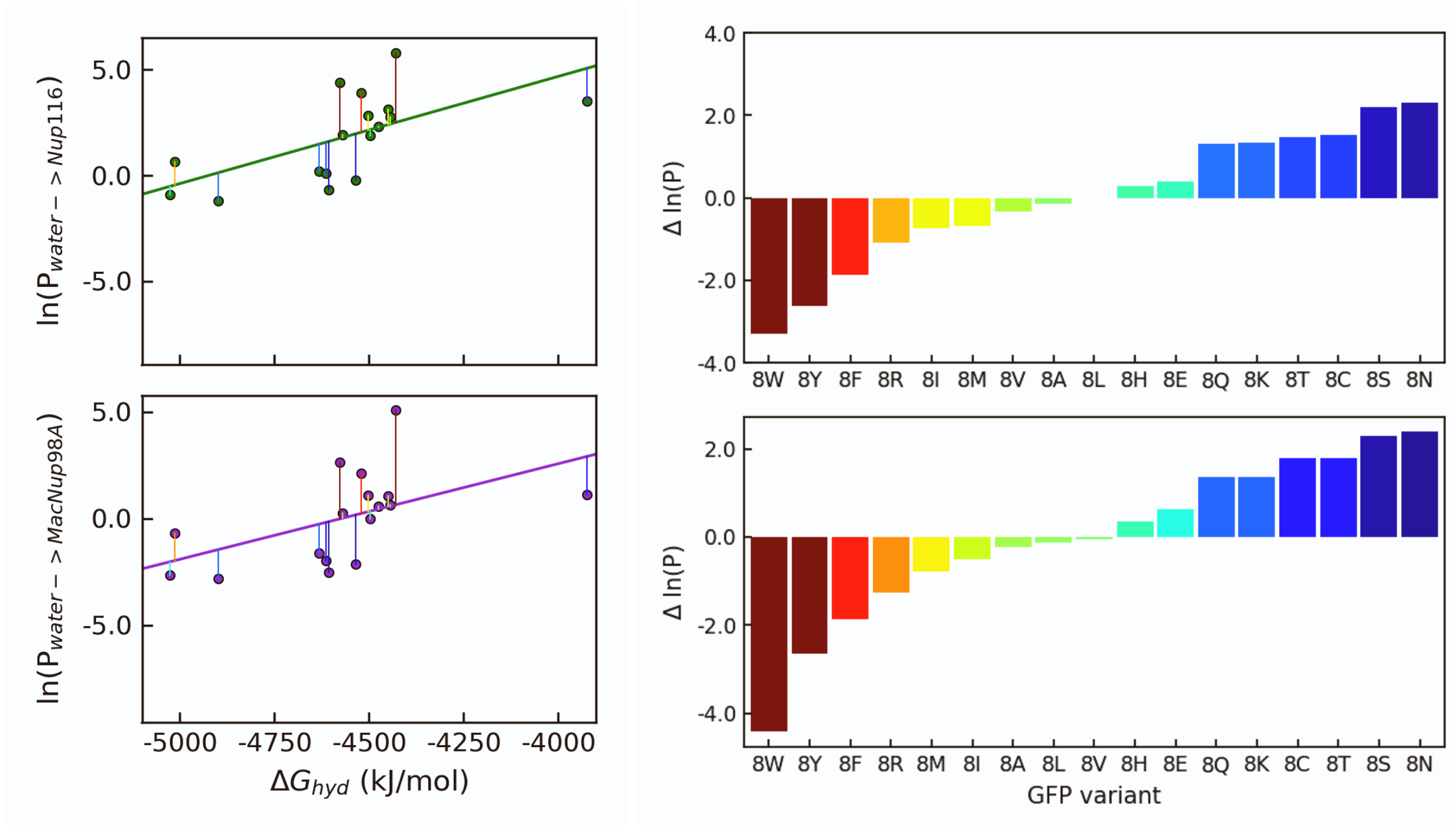

Figure S7: Predictive model and residuals for dynamic implicit solvent simulation trajectory analyzed by hi-patch to calculate hydration free energy.

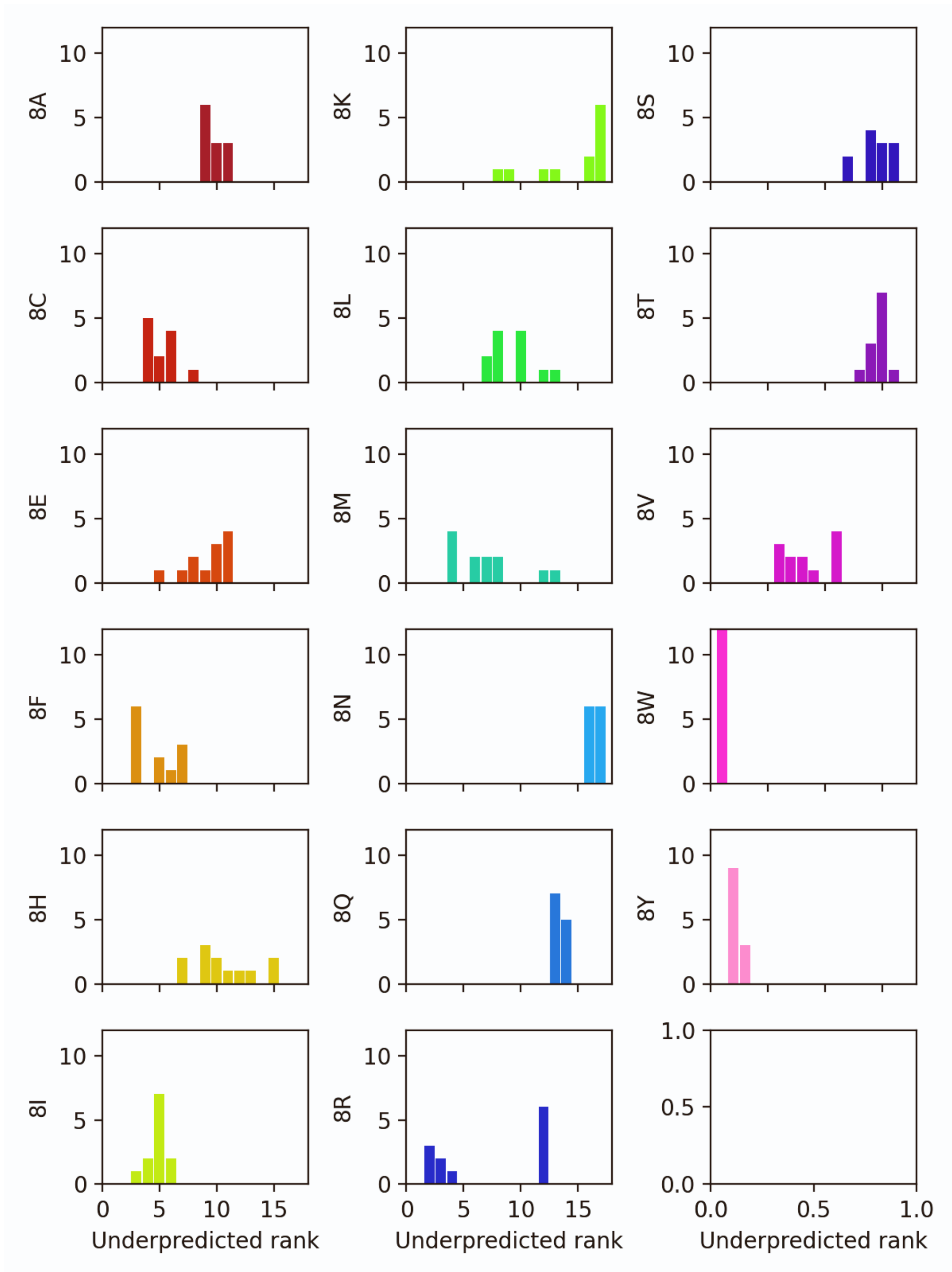

Fig. S8: For all six hydration free energy methods tested, we calculated the ranking of overpredicted and underpredicted proteins and show the histogram of ranks for each GFP variant. Having density toward low numbers indicates underpredictedness, and greater specific interactions between the residue type and scaffold proteins

2. Supporting Tables

Table S1: List of GFP variants from Frey et al.[1] and their amino acid sequences used in modeling of the globular proteins in this work.

Variant	Sequence
**efGFP**	GEELFTGVVPILVELDGDVNGHKFSVRGEGEGDATYGKLTLKFICTTGKLPVPWPTLV<br>TTLTYGVQCFSHYPDHMKQHDFFKSAMPEGYVQERTIYFEDDGAYKTRAEVKFEGDTL<br>VNRIELKGIDFKEDGNILGHKLEYNFNSHNVYIMADKQKNGIKANFNTRHNVEDGSEQ<br>VADHYQQNTPIGDGPVLLPDNHYLNTQSKLSKDPNEKRDHMVLKEFVTAAGI
**8A**	GEELFTGVVPILVELDGDVNGHKFSVRGEGEGDATYGALTLKFICTTGKLPVPWPTLV<br>TTLTYGVQCFSHYPDHMKQHDFFKSAMPEGYVQERAIYFEDDGAYKTRAEVKFEGDTL<br>VNRIELKGIDFKEDGNILGHKLEYNFNSHAVYIMADKQKNGIKAAFNTRHNVEDGSEQ<br>VAAHYQQNTPIGDGPVLLPDNHYLNTASKLSADPNEKRDHMVLKEFVAAAGI
**8C**	GEELFTGVVPILVELDGDVNGHKFSVRGEGEGDATYGCLTLKFICTTGKLPVPWPTLV<br>TTLTYGVQCFSHYPDHMKQHDFFKSAMPEGYVQERCIYFEDDGAYKTRAEVKFEGDTL<br>VNRIELKGIDFKEDGNILGHKLEYNFNSHCVYIMADKQKNGIKACFNTRHNVEDGSEQ<br>VACHYQQNTPIGDGPVLLPDNHYLNTCSKLSCDPNEKRDHMVLKEFVCAAGI
**8E**	GEELFTGVVPILVELDGDVNGHKFSVRGEGEGDATYGELTLKFICTTGKLPVPWPTLV<br>TTLTYGVQCFSHYPDHMKQHDFFKSAMPEGYVQEREIYFEDDGAYKTRAEVKFEGDTL<br>VNRIELKGIDFKEDGNILGHKLEYNFNSHEVYIMADKQKNGIKAEFNTRHNVEDGSEQ<br>VAEHYQQNTPIGDGPVLLPDNHYLNTESKLSEDPNEKRDHMVLKEFVEAAGI
**8F**	GEELFTGVVPILVELDGDVNGHKFSVRGEGEGDATYGFLTLKFICTTGKLPVPWPTLV<br>TTLTYGVQCFSHYPDHMKQHDFFKSAMPEGYVQERFIYFEDDGAYKTRAEVKFEGDTL<br>VNRIELKGIDFKEDGNILGHKLEYNFNSHFVYIMADKQKNGIKAFFNTRHNVEDGSEQ<br>VAFHYQQNTPIGDGPVLLPDNHYLNTFSKLSFDPNEKRDHMVLKEFVFAAGI
**8H**	GEELFTGVVPILVELDGDVNGHKFSVRGEGEGDATYGHLTLKFICTTGKLPVPWPTLV<br>TTLTYGVQCFSHYPDHMKQHDFFKSAMPEGYVQERHIYFEDDGAYKTRAEVKFEGDTL<br>VNRIELKGIDFKEDGNILGHKLEYNFNSHHVYIMADKQKNGIKAHFNTRHNVEDGSEQ<br>VAHHYQQNTPIGDGPVLLPDNHYLNTHSKLSHDPNEKRDHMVLKEFVHAAGI
**8I**	GEELFTGVVPILVELDGDVNGHKFSVRGEGEGDATYGILTLKFICTTGKLPVPWPTLV<br>TTLTYGVQCFSHYPDHMKQHDFFKSAMPEGYVQERIIYFEDDGAYKTRAEVKFEGDTL<br>VNRIELKGIDFKEDGNILGHKLEYNFNSHIVYIMADKQKNGIKAIFNTRHNVEDGSEQ<br>VAIHYQQNTPIGDGPVLLPDNHYLNTISKLSIDPNEKRDHMVLKEFVIAAGI
**8K**	GEELFTGVVPILVELDGDVNGHKFSVRGEGEGDATYGHLTLKFICTTGKLPVPWPTLV<br>TTLTYGVQCFSHYPDHMKQHDFFKSAMPEGYVQERKIYFEDDGAYKTRAEVKFEGDTL<br>VNRIELKGIDFKEDGNILGHKLEYNFNSHKVYIMADKQKNGIKAKFNTRHNVEDGSEQ<br>VAKHYQQNTPIGDGPVLLPDNHYLNTKSKLSKDPNEKRDHMVLKEFVKAAGI

**8L**	GEELFTGVVPILVELDGDVNGHKFSVRGEGEGDATYGLLTLKFICTTGKLPVPWPTLV<br>TTLTYGVQCFSHYPDHMKQHDFFKSAMPEGYVQERLIYFEDDGAYKTRAEVKFEGDTL<br>VNRIELKGIDFKEDGNILGHKLEYNFNSHLVYIMADKQKNGIKALFNTRHNVEDGSEQ<br>VALHYQQNTPIGDGPVLLPDNHYLNTLSKLSLDPNEKRDHMVLKEFVLAAGI
**8M**	GEELFTGVVPILVELDGDVNGHKFSVRGEGEGDATYGMLTLKFICTTGKLPVPWPTLV<br>TTLTYGVQCFSHYPDHMKQHDFFKSAMPEGYVQERMIYFEDDGAYKTRAEVKFEGDTL<br>VNRIELKGIDFKEDGNILGHKLEYNFNSHMVYIMADKQKNGIKAMFNTRHNVEDGSEQ<br>VAMHYQQNTPIGDGPVLLPDNHYLNTMSKLSMDPNEKRDHMVLKEFVMAAGI
**8N**	GEELFTGVVPILVELDGDVNGHKFSVRGEGEGDATYGNLTLKFICTTGKLPVPWPTLV<br>TTLTYGVQCFSHYPDHMKQHDFFKSAMPEGYVQERNIYFEDDGAYKTRAEVKFEGDTL<br>VNRIELKGIDFKEDGNILGHKLEYNFNSHNVYIMADKQKNGIKANFNTRHNVEDGSEQ<br>VANHYQQNTPIGDGPVLLPDNHYLNTNSKLSNDPNEKRDHMVLKEFVNAAGI
**8Q**	GEELFTGVVPILVELDGDVNGHKFSVRGEGEGDATYGQLTLKFICTTGKLPVPWPTLV<br>TTLTYGVQCFSHYPDHMKQHDFFKSAMPEGYVQERQIYFEDDGAYKTRAEVKFEGDTL<br>VNRIELKGIDFKEDGNILGHKLEYNFNSHQVYIMADKQKNGIKAQFNTRHNVEDGSEQ<br>VAQHYQQNTPIGDGPVLLPDNHYLNTQSKLSQDPNEKRDHMVLKEFVQAAGI
**8R**	GEELFTGVVPILVELDGDVNGHKFSVRGEGEGDATYGRLTLKFICTTGKLPVPWPTLV<br>TTLTYGVQCFSHYPDHMKQHDFFKSAMPEGYVQERRIYFEDDGAYKTRAEVKFEGDTL<br>VNRIELKGIDFKEDGNILGHKLEYNFNSHRVYIMADKQKNGIKARFNTRHNVEDGSEQ<br>VARHYQQNTPIGDGPVLLPDNHYLNTRSKLSRDPNEKRDHMVLKEFVRAAGI
**8S**	GEELFTGVVPILVELDGDVNGHKFSVRGEGEGDATYGSLTLKFICTTGKLPVPWPTLV<br>TTLTYGVQCFSHYPDHMKQHDFFKSAMPEGYVQERSIYFEDDGAYKTRAEVKFEGDTL<br>VNRIELKGIDFKEDGNILGHKLEYNFNSHSVYIMADKQKNGIKASFNTRHNVEDGSEQ<br>VADSYQQNTPIGDGPVLLPDNHYLNTSSKLSSDPNEKRDHMVLKEFVSAAGI
**8T**	GEELFTGVVPILVELDGDVNGHKFSVRGEGEGDATYGKLTLKFICTTGKLPVPWPTLV<br>TTLTYGVQCFSHYPDHMKQHDFFKSAMPEGYVQERTIYFEDDGAYKTRAEVKFEGDTL<br>VNRIELKGIDFKEDGNILGHKLEYNFNSHNVYIMADKQKNGIKATFNTRHNVEDGSEQ<br>VATHYQQNTPIGDGPVLLPDNHYLNTTSKLSTDPNEKRDHMVLKEFVTAAGI
**8V**	GEELFTGVVPILVELDGDVNGHKFSVRGEGEGDATYGVLTLKFICTTGKLPVPWPTLV<br>TTLTYGVQCFSHYPDHMKQHDFFKSAMPEGYVQERVIYFEDDGAYKTRAEVKFEGDTL<br>VNRIELKGIDFKEDGNILGHKLEYNFNSHVVYIMADKQKNGIKAVFNTRHNVEDGSEQ<br>VAVHYQQNTPIGDGPVLLPDNHYLNTVSKLSVDPNEKRDHMVLKEFVVAAGI
**8W**	GEELFTGVVPILVELDGDVNGHKFSVRGEGEGDATYGWLTLKFICTTGKLPVPWPTLV<br>TTLTYGVQCFSHYPDHMKQHDFFKSAMPEGYVQERWIYFEDDGAYKTRAEVKFEGDTL<br>VNRIELKGIDFKEDGNILGHKLEYNFNSHWVYIMADKQKNGIKAWFNTRHNVEDGSEQ<br>VAWHYQQNTPIGDGPVLLPDNHYLNTWSKLSWDPNEKRDHMVLKEFVWAAGI

**8Y**	GEELFTGVVPILVELDGDVNGHKFSVRGEGEGDATYGYLTLKFICTTGKLPVPWPTLV<br>TTLTYGVQCFSHYPDHMKQHDFFKSAMPEGYVQERYIYFEDDGAYKTRAEVKFEGDTL<br>VNRIELKGIDFKEDGNILGHKLEYNFNSHYVYIMADKQKNGIKAYFNTRHNVEDGSEQ<br>VAYHYQQNTPIGDGPVLLPDNHYLNTYSKLSYDPNEKRDHMVLKEFVYAAGI

Table S2:Transfer free energies of each GFP into both scaffold proteins calculated from experiments, and hydration free energies from hi-patch. Solvation free energy can be calculated by finding the difference.

Protein	$\Delta F_{tr,\ MacNup98A}$ (kJ/mol)	ΔF_{hyd} (kJ/mol)
8A	-0.7	-3774.2
8C	-2.8	-3810.4
8E	6.6	-4267.8
8F	-5.3	-3660.5
8H	0.0	-3749.6
8I	-2.7	-3796.5
8K	7.0	-4276.2
8L	-1.5	-3777.1
8M	-2.7	-3610.9
8N	6.3	-3870.8
8Q	4.0	-3930.2
8R	1.7	-4200.8
8S	5.3	-3929.4
8T	4.9	-3916.1
8V	-1.6	-3807.2
8W	-12.7	-3616.7
8Y	-6.5	-3723.7

Table S3: List and description of parameters calculated by hi-patch software[3].

# patches	Represents the number of hydrophobic surface regions identified. These patches represent clustered hydrophobic residues exposed to solvent.
Avg. Hydropathy	The mean hydropathy value for each protein variant.
Max. Hydropathy (.mean)	The highest average hydropathy value among all individual surface patches, representing the most hydrophobic surface region for each variant.
Aromaticity	Quantifies the presence of aromatic residues (Tyr, Trp, Phe) on the protein's surface.
Total Patch Solvation ΔG	The total solvation free energy contribution from all identified hydrophobic patches.
Max. Hydropathy (.max)	The single highest hydropathy value at any atomic position on the protein surface, identifying the most hydrophobic individual site for each variant.
Total Hydration Free Energy	The total free energy change (ΔF_{hyd}) when transferring a protein from vacuum to aqueous solution.

Table S4: Sequences for the two Nucleoporin proteins from Frey et al.[1]

**Nucleoporin**	**Sequence**
**MacNup98A**	MFGNTGGGGLFGNTQTQQTGGGLFGQPQQTQFGQTGATGGGLFGGATNTFGGGGG<br>GGLFGGNNNQQTNPTAGGGIFGQGTTGLGGAPAQTGGGLFGAPQNNQGGGLFGGG<br>TTTGGGMFGNQANTQTGGGGLFGGPSQPTTQPPAFSLNNPTTGGGGLFGQPANTM<br>GGNNGGLFGGQTNSFGANNNMLGNNNRPQGAGIFGGATTTAPTGNTGMFGGIGAN<br>NGGGGLFGMNNTNTNPTGGFGATNPTAGGGGLFGGGATTTGGGGLFGGGNTQGGG<br>LLGTANTTAGGLLGGGFNMNNNTGGILGQTNNQFGLGSFGTNNNAAAAPFQPKAS<br>ANGvltkpneknlcyaisngtdfcifelaltqrklvkAGQLKPGAQQAGGMFGQP<br>AQGGNGLFGGGGAATTTPFGGAQNGNLFGGQNTQAQGGGLFGAPVNNAATGAGGG<br>LFGAKPAATTTGGGLFGQMPAQTGGFLGNTATQPAGGGLFGGATTTQAPGGGGGG<br>GLFGGNTTAATTGGGLFGGNTQTGGATGGLFGGQQPNNQGGLFLNTGNANNANTG

	GGLFGGATTTPATGGGLFGGSTNTQPGLATGGGLFGNNQGASQPAAQGGLFGGAA<br>PQQNSLFGGATAGGQTGGLFGGATGATQQQGGGL<br>FGQTASNPTQGGGLFGAANPGLGGAAA
**Nup116**	MFGVSRGAFPSATTQPFGSTGSTFGGQQQQQPVANTSAFGLSQQTNTTQAPAFG<br>NFGNQTSNSPFGMSGSTTANGTPFGQSQLTNNNASGSIFGGMGNNTALSAGSASv<br>vpnstagtsikpfttfeekdpttgvinvfqsitcmpeyrnfsfeelrfqdyqagr<br>kFGTSQNGTGTTFNNPQGTTNTGFGIMGNNNSTTSATTGGLFGQKPATGMFGTGT<br>GSGGGFGSGATNSTGLFGSSTNLSGNSAFGANKPATSGGLFGNTTNNPTNGTNNT<br>GLFGQQNSNTNGGLFGQQQNSFGANNVSNGGAFGQVNRGAFPQQQTQQGSGGIFG<br>QSNANANGGAFGQQQGTGALFGAKPASGGLFGQSAGSKAFGMNTNPTGTTGGLFG<br>QTNQQQSGGGLFGQQQNSNAGGLFGQNNQSQNQSGLFGQQNSSNAFGQPQQQGGL<br>FGSKPAGGLFGQQQGASTFASGNAQNNSIFGQNNQQQQSTGGLFGQQNNQSQSQP<br>GGLFGQTNQNNNQPFGQNGLQQPQQNNSLFGAKPTGFGNTSLFSNSTTNQSNGIS<br>GNNLQQQSGGLFQNKQQPASGGLFGSKPSNTVGGGLFGNNQVANQNNPASTSGGL<br>FGSKPATGSLFGGTNSTAPNASSGGIFGSNNASNTAATTNSTGLFGNKPVGAGAS<br>TSAGGLFGNNNNSSLNNSNGSTGLFGSNNTSQSTNAGGLFQNNTSTNTSGGGLFS<br>QPSQSMAQSQNALQQQQQQR

Table S5: Size, charge and hydropathy[2] of protein sequences used in this work.

Name	Length	Net charge	Urry hydropathy
8A	226	-6	0.573
8C	226	-6	0.575
8E	226	-14	0.552
8F	226	-6	0.581
8H	226	-2	0.579
8I	226	-6	0.577
8K	226	1.5	0.567
8L	226	-6	0.577

8M	226	-6	0.576
8N	226	-6	0.572
8Q	226	-6	0.571
8R	226	2.0	0.571
8S	226	-7.5	0.570
8T	226	-5	0.572
8V	226	-6	0.575
8W	226	-6	0.587
8Y	226	-6	0.583
Nup116	1113	20.5	0.592
MacNup98A	1105	16	0.594

Suporting References

1. Frey, S., R. Rees, J. Schünemann, S. C. Ng, K. Fünfgeld, T. Huyton, and D. Görlich, 2018. Surface properties determining passage rates of proteins through nuclear pores. *Cell* 174:202–217.
2. Urry, D. W., D. C. Gowda, T. M. Parker, C. Luan, M. C. Reid, C. M. Harris, A. Pattanaik, and R. D. Harris, 1992.Hydrophobicity scale for proteins based on inverse temperature transitions. *Biopolymers* 32:1243–1250.
3. Héctor Sánchez-Morán James S. Weltz, D. K. S., and J. L. Kaar, 2021. Understanding Design Rules for Optimizing the Interface between Immobilized Enzymes and Random Copolymer Brushes. *ACS Applied Materials and Interfaces*
4. Lazaridis, T., and M. Karplus, 1999. Effective energy function for proteins in solution. *Proteins: Structure, Function, and Bioinformatics* 35:133–152.
5. Jurrus, E., D. Engel, K. Star, K. Monson, J. Brandi, L. E. Felberg, D. H. Brookes, L. Wilson, J. Chen, K. Liles, et al., 2018. Improvements to the APBS biomolecular solvation software suite. *Protein science* 27:112–128.
6. Mongan, J., C. Simmerling, J. A. McCammon, D. A. Case, and A. Onufriev, 2007. Generalized Born model with a simple, robust molecular volume correction. *Journal of chemical theory and computation* 3:156–169.
