# Supplementary material for: Hydration free energy is an incomplete predictor of globular protein incorporation into condensates
Source: Biophys J. 2025 Dec 4;125(16):4147–56. doi: 10.1016/j.bpj.2025.12.005 (PMC12798646; doi:10.1016/j.bpj.2025.12.005)
Supplement: Supplementary file 2 [file mmc2.pdf]

Biophysical Journal
Article

Biophysical Society

Hydration free energy is an incomplete predictor of globular protein incorporation into condensates

Sophie Anderson,[1] Malcolm Harrison,[2] and Gregory L. Dignon[2,*]
[1]Department of Biochemistry, Vassar College, Poughkeepsie, New York and [2]Department of Chemical and Biochemical Engineering, Rutgers University, Piscataway, New Jersey

ABSTRACT Membraneless organelles (MLOs) are assemblies of biomolecules that function without a dividing lipid membrane in a cellular environment. These MLOs, termed biomolecular condensates, are commonly formed by the thermodynamic process of liquid-liquid phase separation and assembly of large numbers of proteins, nucleic acids, and co-solvent molecules. Within MLOs, certain biomolecule types are particularly causative of phase separation and are termed “scaffolds” as they provide the major driving forces for self-assembly. Other molecules that are present in a condensate but are less causative than the scaffold molecules are termed “clients.” Much effort has recently decoded many of the molecular interactions underlying liquid-liquid phase separation in search of predicting equilibrium concentrations and materials properties of condensates. In this work, we provide a simple computational approach that may predict the partitioning of globular protein clients into condensates primarily composed of disordered protein scaffolds. Specifically, we use multiple methods to calculate hydration free energy of a series of globular green fluorescent protein variants and find that hydration free energy is relatively well-correlated with the partition coefficient of these proteins into FG nucleoporin condensates. We then provide a comparison of different hydration free energy predictors and discuss why some may provide a more accurate prediction of partitioning. Finally, we discuss the shortcomings of hydration free energy as a predictor by identifying other possible confounding factors such as specific interactions, charge matching, and differential solvation inside a condensate, which will aid in making more robust predictions in future studies trained on more diverse data sets.

SIGNIFICANCE Understanding the extent to which molecules can partition into biomolecular condensates is crucial for deciphering cellular organization and function. This study introduces a simple computational approach to predict the partitioning of globular GFP proteins into condensates comprising FG nucleoporins using hydration free energy calculated from static structures of GFP as a key predictor. By comparing different hydration free energy predictors, we highlight the limitations of relying solely on hydration free energy, emphasizing the need to consider other molecular factors. We finally analyze the shortcomings of the hydration free energy predictions and discuss other factors that contribute to partitioning of clients into condensates, namely specific interactions and net charge of the scaffold molecules, and different properties of water in the condensate.

INTRODUCTION

Biomolecular condensates, or membraneless organelles (MLOs), are cellular compartments made up of self-assembled biomolecules that lack a dividing lipid membrane as is present in membrane-bound organelles (1,2). Many biomolecular condensates are formed through the process of liquid-liquid phase separation (LLPS), in which a homogeneous solution of macromolecules separates into two or more liquid-like phases having distinct molecular compositions (3–5). The self-association of molecules involved in driving LLPS is predominantly due to weak multivalent interactions between proteins and nucleic acids, associating with each other to form a “dense” phase and isolating from the surrounding “dilute” phase (6–8). The interior of a condensate differs from that of its surroundings through various emergent properties including surface tension, viscoelasticity, and differential solvation of small molecules (9,10). Biomolecular condensates coordinate and execute specific

Submitted July 3, 2025, and accepted for publication December 1, 2025.

*Correspondence: gregory.dignon@rutgers.edu
Sophie Anderson’s present address is Department of Epidemiology Columbia University, New York, New York 10032.
Sophie Anderson and Malcolm Harrison contributed equally to this work.

Editor: Ana Nicoleta Bondar.
https://doi.org/10.1016/j.bpj.2025.12.005

© 2025 The Author(s). Published by Elsevier Inc. on behalf of Biophysical Society.
This is an open access article under the CC BY license (http://creativecommons.org/licenses/by/4.0/).

functions integral to various biological processes, including DNA repair, cellular stress response, filtration, and signal transduction (11–14). Dysregulated biomolecular condensates are associated with the development and progression of cancer, as well as neurological diseases such as ALS (15–18).

Most MLOs contain dozens to hundreds of types of molecules, including proteins, nucleic acids, small molecules, and solvent (19–23). Molecules that are particularly causative of phase separation through attractive interactions are termed scaffolds, whereas other components that are less causative, but still incorporate into condensates, are termed clients (4,21,24). The identity and materials properties of a condensate are largely linked to the composition and concentration of molecular components and their interconnectedness. Additionally, the role of hydration in condensates has been recently gaining more interest, showing that the water content inside condensates and behavior of surface-interacting water and released water can influence phase separation of proteins (25–27). Therefore, it is essential to have a good understanding of what driving forces control composition of condensates for characterization of condensate function and materials properties and for design of new clients and condensate-interacting molecules (4,22,24,28).

Many studies have helped decode the molecular grammar of phase separation, particularly focusing on sequence and composition of intrinsically disordered regions, which are common in phase-separating proteins (29–34). However, many condensates include partially folded or completely folded globular proteins, which cannot be fully described based on their amino acid sequence (35,36). It is useful to establish simple and generalizable metrics to quantify and predict the partitioning of globular proteins into condensates, as this enables a better understanding of the molecular determinants that govern condensate composition and function, and it facilitates the development of predictive models for biological and biomedical applications.

Multicomponent phase separation can occur through several distinct modes, including cooperative co-phase separation, scaffold-client co-phase separation, cross-interaction-driven co-phase separation, and exclusive phase separation (4,28,37,38). Among these, scaffold-client systems are particularly widespread and experimentally tractable, making them valuable models for investigating the factors that influence client protein incorporation (19,22,39). However, the highly dynamic and disordered nature of condensate interiors presents challenges in identifying which specific protein interactions are most important for phase separation and client partitioning. Therefore, development of predictive metrics can aid in elucidating the underlying physical principles that determine condensate composition and inform future studies of protein behavior in these environments.

In this work, we derive a thermodynamic cycle that relates hydration free energy to the transfer free energy of green fluorescent protein (GFP) variants partitioning into FG nucleoporin condensates, providing a quantitative model for scaffold-client systems. We assess the correlation between the experimentally measured partition coefficients (P) of GFP variants and their computed hydration free energies (ΔF_{hyd}), finding that hydration free energy is a strong predictor of partitioning behavior within this system. We then systematically evaluate several computational models for estimating hydration free energy, finding that static structure-based approaches such as the hydrophobic intensity patch (hi-patch) method and effective energy function 1 (EEF1) are decently predictive of experimental partition coefficients while being computationally inexpensive and relatively easy to use. Finally, we conduct an analysis of possible sources of deviation from these predictions, highlighting additional factors such as the scaffold molecule's physicochemical properties and specific scaffold-client interactions, each of which have an influence on partitioning.

MATERIALS AND METHODS

For each GFP protein variant, eight residues on the surface of the protein were mutated to the same amino acid. For example, GFP variant 8A contains eight surface mutations, all of which were converted to the amino acid alanine. The same eight regions of the amino acid sequence were modified on each of the 17 GFP variants, according to the model system variants. The sequences were obtained from Frey et al. (20) and are listed in Table S1. To generate structures of the 17 designed GFP sequences, we used AlphaFold3 to generate PDB structures, which were then energy minimized (40). We directly used these minimized structures for static ΔF_{tr} calculations and as starting points for dynamic methods.

The EEF1 of CHARMM was used as it calculates the ΔF_{hyd} of all atoms without considering the surface area (41). The adaptive Poisson-Boltzmann solver (APBS) was used because it solved the Poisson-Boltzmann equation of the protein and vacuum to approximate the change in ΔF_{hyd} (42). The hi-patch method was adapted to report the hydration free energy (43). The original implementation reports only hydrophobicity of hydrophobic patches on the surface of the protein, whereas we report hydration of the full protein surface. The calculations are based on of the EEF1 implicit solvent model (41) and corrected for effects of neighboring atoms, highlighting the cooperative effects of nonpolar atoms in creating hydrophobic interfaces with water (44–46).

Molecular dynamics (MD) simulations were performed with OpenMM in both implicit and explicit solvent setups (47). We utilized Amber ff14SB for all simulations (48). Implicit solvent simulations were conducted in the NVT ensemble using the GBNeck implicit solvent model (49). Explicit solvent simulations were initially equilibrated in the NVT ensemble, followed by production runs in the NPT ensemble. Temperature was maintained at 300 K using the Langevin middle integrator, and pressure was kept constant in explicit simulations using a Monte Carlo barostat. All simulations were conducted at 0.1 M salt concentration and were carried out for 100-ns simulation time. The first 20 ns were discarded for equilibration time.

RESULTS AND DISCUSSION

Hydropathy of surface-decorated residues predicts surface mutation effects on partitioning

As a model system, we use data from Frey et al., who developed a series of GFP variants with the same folded structure but different surface properties. Namely, they mutated eight

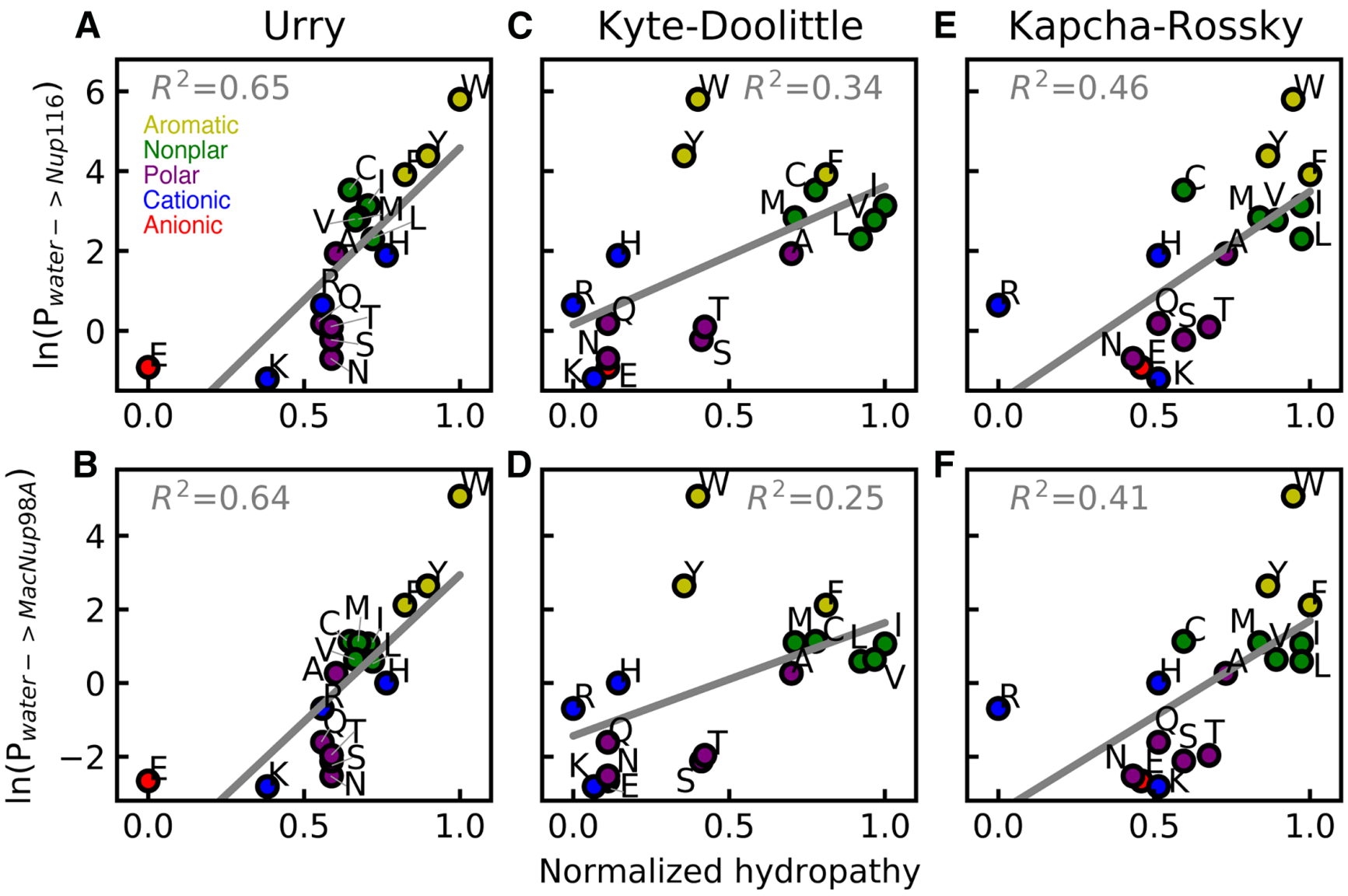

FIGURE 1 Correlation of ln(P) into two separate Nup condensates with three hydropathy scales: (*A* and *B*) the Urry hydropathy scale, (*C* and *D*) the Kyte-Doolittle scale, and (*E* and *F*) the Kapcha-Rossky scale. Quality of fit is shown by R^2 values in each plot.

sites on the surface of the protein, all to one particular amino acid, for 17 of the 20 canonical amino acids (Table S1). Using two different FG nucleoporin proteins (FG Nups; Nup116 and MacNup98A) as scaffolds, they quantified the partitioning of the 17 GFP variants into condensates of each scaffold, resulting in 34 total measurements of partition coefficients (20). We can convert these measurements of partition coefficients to transfer free energies as $\Delta F_{tr} = -k_B T \ln(P)$. We find that the transfer free energy of the globular proteins into condensates can vary from most incorporated −14.5 kJ/mol (8W) to most excluded, 7.0 kJ/mol (8K) at 298 K (Table S2). Within this data set, all protein variants have the same eight sites mutated, which enables us to ask how well a simple hydropathy scale can predict the difference between each variant's partitioning behavior.

Taking the log of the experimental partition coefficients as a proxy for ΔF_{hyd}, we show the correlation of the experimental data with a few popular hydropathy scales (Fig. 1). The value used from each scale is simply a single value for the amino acid that is decorated on the surface for the corresponding variant. We find that the best correlation comes from using the Urry scale, which is directly derived from experimental measurements of protein phase separation (Fig. 1 *A* and *B*) (50). The Kyte-Doolittle scale has the poorest agreement with the experimental data (Fig. 1 *C* and *D*), likely because it is derived from experiments measuring the solubility of the amino acids and does not account for amino acids in context of a protein or condensate (51). Finally, the Kapcha-Rossky hydrophobicity scale, which was derived from partial charges on atoms in each amino acid, achieves intermediate predictive capability (Fig. 1 *E* and *F*) (52). The quality of fit between hydropathy models is in line with other work showing that of these hydropathy scales, the Urry scale gives the best agreement with experimental phase separation data when used in coarse-grained MD simulations (53,54). These results are also consistent with calculations by Villegas et al. for Kyte-Doolittle and Kapcha-Rosssky applied to the same system (55).

Thus, using the Urry hydropathy scale could potentially serve as an approach to predict the change in partitioning of a globular protein upon mutations to its surface. However, as a general predictor of globular protein partitioning, the Urry hydropathy scale does not account for the remaining surface residues of the GFP molecules. Previous work from Villegas et al. has developed statistical potentials for each amino acid and its "stickiness" or solubility, and interaction potential, showing exceptional agreement and predictability of these data (55). Therefore, we next analyze the full surface of the globular GFP variants and assess what surface features are most important to predict partitioning into nucleoporin condensates.

Thermodynamic cycle of solvation and hydration

Hydration free energy (ΔF_{hyd}) is a measure of the change in free energy of a molecule from vacuum to pure water solvent (Fig. 2) and will generally be negative for soluble molecules (56). Hydration free energy is commonly approximated by the transfer from oil to water, since oil represents a hydrophobic environment (57). Similarly, the transfer free energy of a molecule partitioning into a condensate can be mathematically defined as the difference between the free energy of a molecule inside and outside a condensate and is logarithmically related to the molecule's partition coefficient (P):

$$\Delta F_{tr} = F_{inside} - F_{outside} = -k_B T \ln(P), \quad (1)$$

where $P = c_{cond}/c_{sat}$ is the ratio of a molecule's concentration inside the condensate (c_{cond}) to its concentration outside

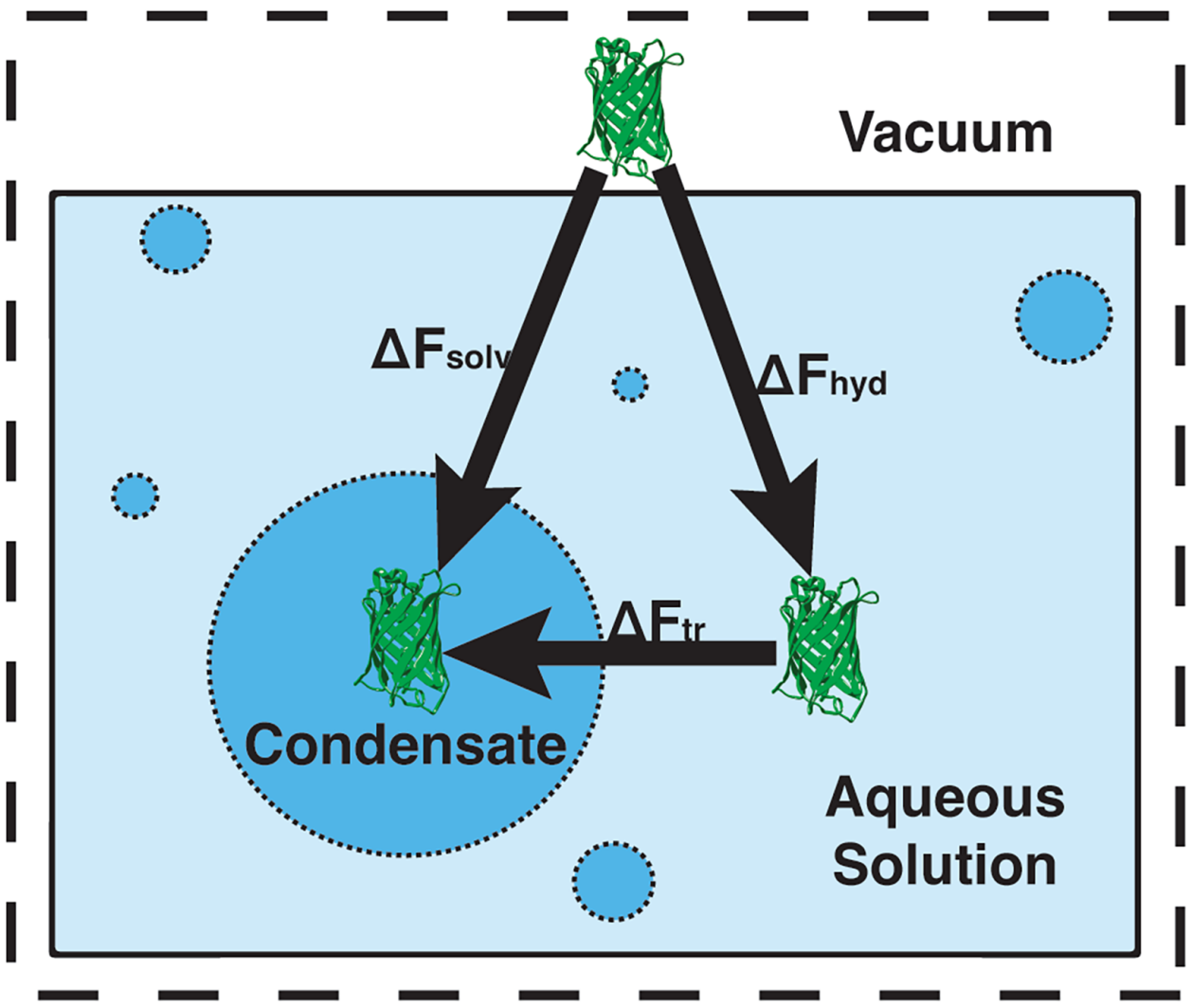

FIGURE 2 A simple thermodynamic cycle to describe transfer free energy of protein between vacuum, low concentration aqueous solution, and condensate environments.

the condensate (c_{sat}). For scaffolds, this tends to be a large number on the order of tens to thousands, whereas for clients, it can be more moderate or even less than 1 for a molecule that is excluded from a condensate. Notably, in multicomponent systems, the partition coefficient and saturation concentration are dependent on the total overall concentration of each component in the system (4,24,58). Various experimental methods are capable of measuring partition coefficients and thus can be used to calculate transfer free energy of a client protein into a condensate composed of a separate scaffold protein (20,23). In experiments where the condensate is primarily composed of scaffold, and the external phase has a very low scaffold concentration, there should be minimal effect on the client's partition coefficient, as it is similar to the scenario described by the definition of ΔF_{tr}.

Notably, this ΔF_{tr} represents the free energy required to transfer between two aqueous phases, specifically between aqueous surroundings and a water-containing protein-rich condensate, and it could be expected to differ significantly from oil-water transfer free energy of the same molecule. However, recent work has shown that oil-water transfer is a good predictor of small-molecule partitioning into condensates, perhaps due to the differential solvent behavior of water inside a condensate (10). To fully describe the transfer free energy of a protein into a condensate and relate it to hydration free energy, we devise a thermodynamic cycle, distinguishing between transfer from vacuum to pure water, vacuum to condensate, and the difference between these two being equivalent to the transfer free energy (Fig. 2).

$$\Delta F_{tr} = \Delta F_{solv} - \Delta F_{hyd} = -k_B T \ln(P) \quad (2)$$

We note that the value of ΔF_{solv} is more challenging to obtain directly but can be calculated indirectly through the thermodynamic cycle if ΔF_{tr} and ΔF_{hyd} are determined individually. This value would indicate the free energy change upon insertion to a condensate from vacuum, which is of little practical value. Thus, we focus primarily on the relation between ΔF_{hyd} and ΔF_{tr} for the remainder of this paper and only invoke ΔF_{solv} to explain deviations in the data. Notably, values of ΔF_{tr} are of much smaller magnitude than ΔF_{hyd} or ΔF_{solv} due to the similarity of water to condensate interior as compared with vacuum.

Relation between hydration free energy and partition coefficients of GFP variants

To approximate the ΔF_{tr} of each GFP variant into the Nup condensates, we calculated the ΔF_{hyd} using static structures of each globular GFP variant using hi-patch. Hi-patch is a method designed to identify hydrophobic patches on protein surfaces, based on parameters from the EEF1 implicit solvation model, and correcting each atom's hydration based on its neighboring atoms (41,43). This approach is motivated by observations that hydropathy is influenced not only by presence of nonpolar atoms but also by patchiness of nonpolar atoms, having many within close proximity to each other (44,45). The hi-patch method calculates multiple surface geometry-based features including number of hydrophobic patches, the surface area of each patch, and the average hydropathy of each patch, among others (Fig. S1; Table S3). We find that of these properties, the absolute hydration free energy, ΔF_{hyd}, is the best predictor of ΔF_{tr} (Figs. 3 and S3). We find that more negative values of ΔF_{hyd} correspond to greater partitioning of the client into the condensate, highlighting an inverse correlation between ΔF_{hyd} and ΔF_{tr}. For individual residue types (e.g., polar), this relationship seems to hold rather well. One notable deviation from expected behavior is for aromatic amino acids where phenylalanine (F) is more hydrophobic than tyrosine (Y) according to our calculations but is less incorporated into condensates than Y according to experiments (20). This is in line with recent findings by de Sancho and López (59). Notably, the results seem to disagree with well-established studies of L-phenylalanine and L-tyrosine solubility, where tyrosine tends to be significantly less soluble than phenylalanine (60). These discrepancies could be explained by the context of amino acids, presenting primarily their side chain to solution and disrupting the tight packing of tyrosine into crystals, which is not possible when within a protein.

This is reasonable because the condensate environment, while still aqueous, has a smaller water content than the exterior, making more soluble proteins prefer the aqueous exterior to the condensate interior. Notably, the hydration free energy of only the hydrophobic patches on the protein surface is also a reasonable predictor for the partition coefficients, as well as the total number of patches and the aromaticity of the surface (Fig. S2). Of these, the

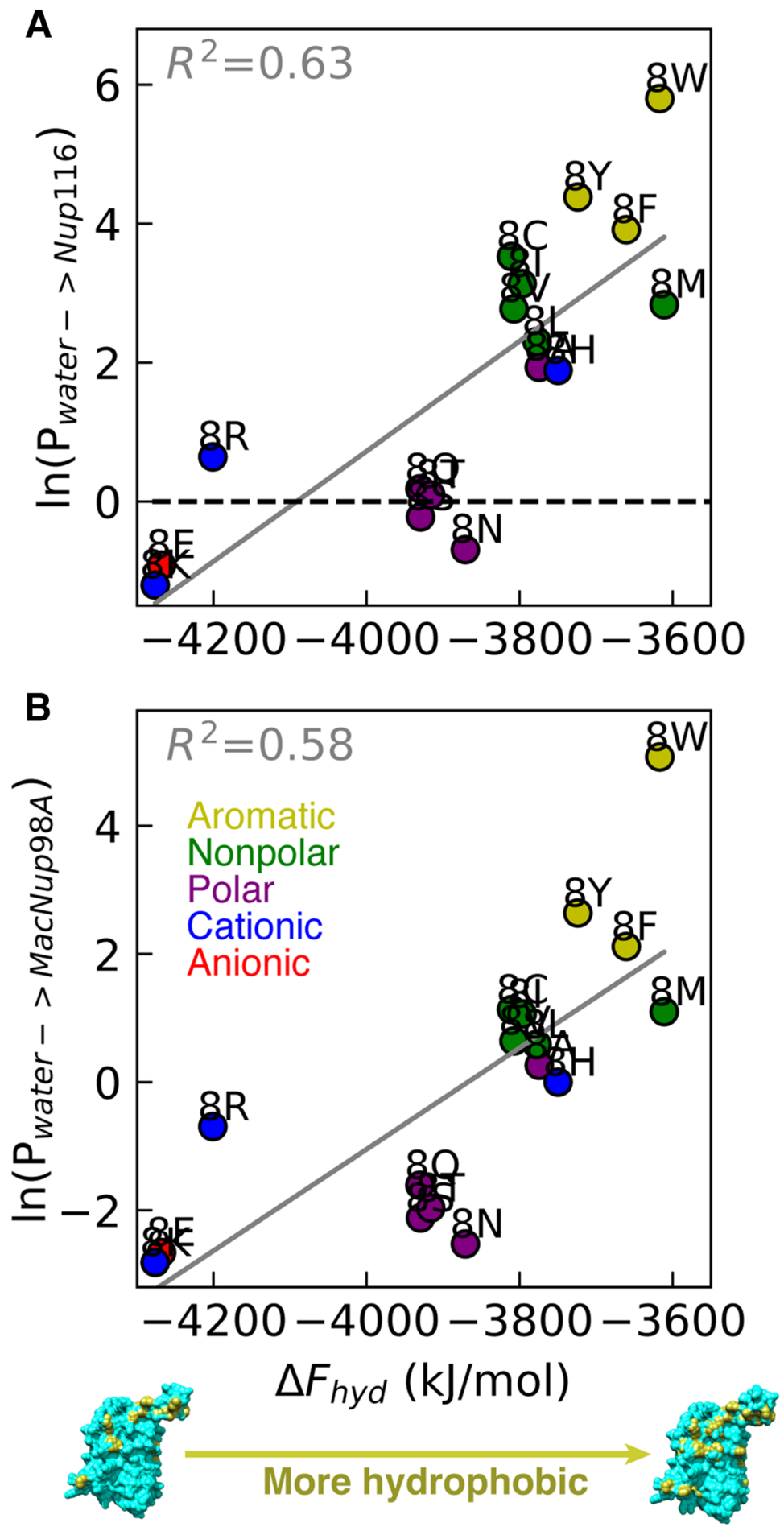

FIGURE 3 Correlation of computationally calculated ΔF_{hyd} and experimentally measured partition coefficient for 17 variants of GFP into (*A*) Nup116, and (*B*) MacNup98A. Renderings show least hydrophobic and most hydrophobic GFP variants visualized using hi-patch per-atom hydropathy output.

aromaticity metric is the least redundant with total hydration free energy, having a covariance of 0.6 with total ΔF_{hyd} (Fig. S1). Such additional features may be added to the predictive model but risk overfitting the model in absence of a larger training data set.

Thus, we conclude that predicted ΔF_{hyd} of a protein is a decent predictor of partitioning of a globular protein into a condensate but does not account for all factors. Another consideration is that the relationship between ΔF_{hyd} and ln(P) is quantitatively different between Nup116 and MacNup98A. Although the ΔF_{hyd} of each globular protein is independent of the scaffold molecule, the partition coefficients will usually be different, thus resulting in different quantitative fits. Since Nup116 is longer than MacNup98A, it has greater valency and number of aromatic residues (Table S4), and thus, it stands to reason that the clients would be more prone to partition into their condensates due to greater interaction propensities with the scaffolds (61). Within this data set, the two scaffold molecules have relatively similar composition (Tables S4 and S5), so there is not a very large difference in partitioning of clients into these condensates. It is plausible that partitioning of these same clients into condensates of compositionally distinct scaffold proteins would result in more significant deviation from this behavior (22,62).

Comparison of hydration models for predicting partitioning

Although the hi-patch method provides decent predictive capabilities of GFP partitioning into Nup condensates, we ask the question whether other hydration models are more appropriate for this problem. Thus, we carried out ΔF_{hyd} calculations using two additional static methods, applied directly to static structures, as well as three dynamic approaches that carry out MD simulations of the protein to capture fluctuations of the structure and surface of the protein.

Table 1 shows the different hydration models used and the correlation to the log of the experimentally measured partition coefficients. The models chosen for these ΔF_{hyd} calculations all vary in how they approximate solvation free energy and what they include or if they neglect to balance accuracy with computational power. CHARMM EEF1 is a great base for calculating ΔF_{hyd}, and in the 25 years since its publication, several advancements in computation speed have allowed for more accurate approximations, such as fast surface area calculations and the ability to use environmental corrections and account for neighboring atoms affecting solubility, as in the updates applied in the hi-patch method (41,63). The APBS model does not explicitly account for ion-solvent interactions and models the solvent as a dielectric continuum, which can affect the accuracy of the approximate hydration free energy (42). The GBNeck model approximates the generalized Born implicit solvent model to be able to obtain a solution faster for use with MD simulations, which may result in errors from the true value of the change in ΔF_{hyd} (49).

To assess the effect that dynamic structure change has on ΔF_{hyd}, MD simulations were performed using both the GBNeck implicit solvent model as well as explicit solvent simulations in TIP3P water (48). We directly calculated

TABLE 1 Quality of agreement Between different hydration models and experimental partition coefficients

Method	Input	Nup116 R^2	MacNup98A R^2
Hi-patch	static	0.6334	0.5752
CHARMM EEF1	static	0.3981	0.3166
ABPS	static	0.1289	0.1185
GBNeck	dynamic	0.1015	0.0919
Hi-patch traj explicit	dynamic	0.4759	0.4154
Hi-patch traj implicit	dynamic	0.3815	0.2721

The hi-patch algorithm, CHARMM EEF1, and APBS were applied to static structures. Note that GBNeck and hi-patch traj implicit methods are applied to the same trajectory, just using different hydration models.

the hydration free energy of the GFP molecules from GBNeck as reported by the implicit solvent model and calculated the average over the trajectory. This resulted in the poorest prediction of experimentally measured partition coefficients. We thus applied the hi-patch algorithm to the trajectory generated by both the GBNeck implicit solvent model and the explicit solvent simulation, taking the average of all frames and finding that it provided better predictions for experimental partition coefficients. This indicates that the APBS and GBNeck hydration models struggle to report qualitatively accurate values for hydration free energy between different similar proteins, whereas EEF1 and hi-patch provide the most useful qualitative predictions.

Interestingly, the hi-patch method provided the best predictions when applied to only a static structure and weaker predictions when applied to a dynamic trajectory from MD simulations. The reason for this could be due to weaknesses in the conformational sampling of the MD simulations or perhaps due to different populations of states occurring within the condensate that would not be accounted for in the bulk aqueous MD simulations. Out of all of the static and dynamic methods used to predict ΔF_{hyd}, hi-patch had the strongest linear correlation between ΔF_{hyd} and ln(P) (Fig. 3; Table 1). Furthermore, the hi-patch calculation can be carried out nearly instantaneously and only requires a static 3D structure of the protein, making it accessible to implement without need for expertise in computational research or significant computing power. It should be noted that the most common applications for APBS and GBNeck are for relative binding free energy of ligands to proteins and application to MD simulations, respectively, and thus, they are not purposed for calculating absolute solvation free energies of proteins (49,64,65).

It may be beneficial to attempt more rigorous quantitative calculations of hydration free energy (56,66). However, it is unclear whether the benefits of additional accuracy will be sufficient to account for significantly larger computational cost. Future work could evaluate additional methods but would benefit from a larger and more diverse set of globular client proteins as a training set. It should be noted that previous work has achieved greater prediction of partitioning of GFP variants into nucleoporin condensates using a statistical potential derived from protein structures (55). This work, rather than deriving a hydration free energy, derives a "stickiness" that quantifies the transfer free energy of each amino acid residue from aqueous solvent to a typical protein interface. Similar approaches have quantified hydration and transfer free energies of amino acids using atomistic simulations (59,67). From the current data set, since we are applying hydration free energy calculations, we are able to observe the shortcomings of neglecting the protein-rich environment of a condensate, and we hypothesize possible causes for error in the predictions from hi-patch-calculated ΔF_{hyd}.

Specific interactions and differential solvation driving partitioning

We hypothesize three potential sources of error from our predictions as 1) specific interactions between the surface residues of GFP and the residues in the scaffold protein, 2) the matching of charges in the condensed phase, and 3) the altered solvent environment inside the condensate (10,62,67). To explore this, we compute the error of predictions from the logarithmic fit between ΔF_{hyd} and P from experimental results as ΔF_{tr} Error $= RT \ln(P_{actual} - P_{predict})$ (Fig. 4).

Related to the question of specific interactions, we find that GFP variants decorated by tyrosine (Y), tryptophan (W), and arginine (R) residues are systematically underpredicted by our hydration-based model (Fig. 4). These residues, particularly R and Y, have been identified as highly causative of phase separation in disordered proteins (29,68) owing to their sticker-like behavior in the theory of associative polymers (69,70). This can be attributed to their unique interaction propensities since Y and W are aromatic and likely engage in π-π stacking with aromatic groups in FG nucleoporins, whereas R is known to participate in strong cation-π interactions with aromatic residues (29,68,71). The guanidinium group of R is especially effective at such interactions, as it can simultaneously form hydrogen bonds and cation-π interactions, which may account for the greater underprediction of its partitioning relative to other positively charged residues (68). These systematic deviations suggest that although hydration free energy captures a large part of the partitioning behavior, fully accurate models will likely require explicit consideration of these more specific physicochemical interactions, such as has been used in various coarse-grained models (72,73). We note that the amino acid composition, and particularly the aromatic fraction of both scaffold molecules, is similar (Tables S4 and S5), so the extent of specific interactions confounding the purely hydration free energy-based prediction is likely to be similar between both cases. As such, we find both Nup116 and MacNup98A partition coefficients are underpredicted to similar degrees for each of these amino acids. We also find that W and Y are the most underpredicted clients for all hydration models tested in this work (Figs. S3–S8), whereas R is underpredicted by about half of the methods. This can likely be explained by differences in hydration methods' handling of formally charged amino acids. We also note that phenylalanine (F) is generally well-predicted by most models even though it is aromatic and can participate in specific interactions with aromatic residues in the scaffold. The fact that it is not underpredicted can be explained by recent findings showing that Y, whereas less hydrophobic than F, tends to transfer

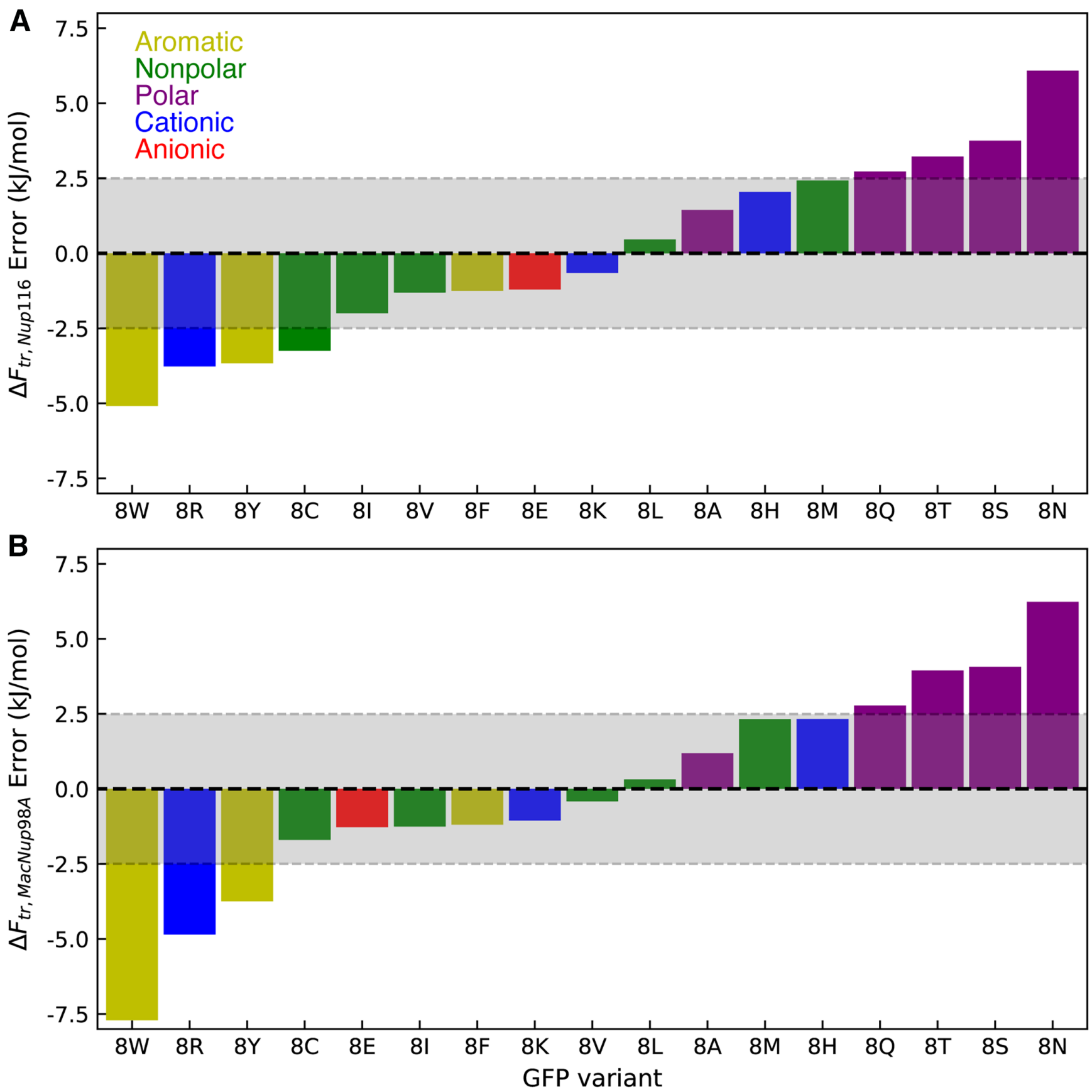

FIGURE 4 Sorted ΔF_{tr} values comparing predicted partitioning, to experimental measurements for (*A*) Nup116 and (*B*) MacNup98A. Sorting was done to display order of most underpredicted to most overpredicted. Gray boxes are drawn around values of ± 1 k_BT.

into typical condensates more strongly than F due to the preference of F for more hydrophobic condensates (59).

Charge matching between the client and scaffold is also likely to influence partitioning of globular clients into condensates. Both scaffold molecules carry a net positive charge, and thus, we would expect there to be a preference for negatively charged clients to partition into the condensate. In our data, we observe that positively charged lysine (K) variant is more excluded from the condensate than the negatively charged glutamate (E) variant, despite glutamate's high solubility. We also note that E ranks lower than K in being underpredicted for all hydration free energy methods tested (Figs. S3–S8). This supports the notion that charge matching is an important factor of condensate partitioning that is not captured by calculations of hydration free energy. Although arginine also carries a positive charge, and should thus have some tendency to be excluded from the Nup116 and MacNup98A droplets, we find that it tends to be underpredicted in most cases, but that is likely due to specific interactions with aromatic residues and sp^2-hybridized groups in the condensate.

Another set of amino acids that are poorly predicted in most cases are the polar uncharged amino acids Q, T, S, and N. One possible explanation for this is highlighted in previous work that finds that small-molecule partitioning into condensates is most well predicted by oil-water transfer (10). This indicates that the condensate interior behaves more hydrophobic than expected for a predominantly aqueous environment, which may contribute to the overprediction of globular clients decorated with polar uncharged amino acids. Since these residues are soluble in water, their respective GFP variants are already predicted to have a relatively low partitioning into the condensates. However, their partitioning was overpredicted, indicating that their presence inside the condensate is even less favorable than predicted by simply just hydration free energy and the reduced water content inside the condensate. This could be evidence that water inside the condensate behaves differently than bulk water and is a more favorable environment for hydrophobic molecules, rather than polar molecules. This suggests that the microenvironment inside protein-rich condensates can deviate substantially from bulk solvent properties, though this remains a speculative explanation.

Further insight is gained by comparing the ranking order of GFP variants across experimental data and computational models (Fig. S8). The W variant is consistently ranked as the most underpredicted variant, whereas the aromatic-rich and arginine-containing variants (R, Y, F) are at least 50% among those with the greatest degree of being

underpredicted. In contrast, the polar variants (N, Q, S, T) are consistently ranked lowest, or the most overpredicted. Charged variants display more variable behavior across models, reflecting the differing abilities of computational models to capture charge effects. These observations underscore both the strengths and limitations of hydration-based models and emphasize the need for further refinement to account for specific interactions and environmental effects. Future studies should directly incorporate these additional effects of charge matching, specific interactions, and altered solvent environment to more accurately and robustly predict client partitioning into condensates and will require a more diverse experimental data set.

CONCLUSION

Our results demonstrate that hydration free energy is a strong predictor of the partitioning behavior of GFP variants into FG nucleoporin condensates. Across 17 GFP variants, we observed a robust logarithmic correlation between computed hydration free energy and experimentally measured partition coefficients, supporting the use of hydration free energy as a proxy for transfer free energy in these systems. Variants that are underpredicted by the model are enriched in amino acids capable of specific interactions with aromatic residues of the FG nucleoporins, such as tyrosine, tryptophan, and arginine. This underprediction is likely due to π-π and cation-π interactions that are not fully represented by ΔF_{hyd} calculations alone. Polar variants are generally overpredicted, suggesting that the condensate interior may be less accommodating to polar side chains due to reduced polarity or solvation capacity. Among the computational methods evaluated, the hi-patch model provides the best agreement with experimental data, indicating that detailed analysis of surface features is critical for accurate prediction of partitioning behavior. This work establishes an approach for predicting scaffold-client phase separation and partitioning behaviors of proteins into condensates, which can be applied to generate quick rough estimates of the general partitioning capability of different globular proteins. This case study highlights the plausibility of hydration free energy as a general predictor of partitioning behavior, particularly into condensates generally, though its predictive power may fall short of other methods that account directly for hydration and transfer free energy of amino acid residues into condensates (55). The strength of this approach is to be agnostic of condensate interior properties and only look at the impact of solubility on protein partitioning and how far it can be taken to predict phase separation propensity. As techniques are being developed and improved to aid in the quantification of distinct molecular concentrations inside condensates (23), as well as the behavior of water in condensates (26,27), we anticipate future work will enable more rigorous validation of the general principle highlighted in this work.

DATA AND CODE AVAILABILITY

Raw data for figures, PDB files and computational workflows to reproduce results have been deposited at Zenodo under the accession number https://doi.org/10.5281/zenodo.17494616 and are publicly available as of the article publication date.

ACKNOWLEDGMENTS

The authors would like to thank Ben Schuster, José Villegas, Emiliano Brini, and Pradipta Bandyopadhyay for helpful conversations. We also thank Héctor Sánchez-Morán and Joel Kaar for useful conversations and assistance with the hi-patch code. G.L.D. acknowledges support from NIH NIGMS award number R35GM150589 and start-up funds from Rutgers University. S.A. was supported through the Advanced Materials REU summer fellowship at Rutgers University through NSF award DMR-2149971.

AUTHOR CONTRIBUTIONS

G.L.D. designed the research. S.A. and M.H. performed simulations and analyzed the data. S.A., M.H., and G.L.D. wrote the article.

DECLARATION OF INTERESTS

The authors declare no competing interests.

DECLARATION OF GENERATIVE AI AND AI-ASSISTED TECHNOLOGIES IN THE WRITING PROCESS

During the preparation of this work the authors used Perplexity in order to draft one short section of the paper. After using this tool/service, the authors reviewed and edited the content as needed and take full responsibility for the content of the publication.

SUPPORTING MATERIAL

Supporting material can be found online at https://doi.org/10.1016/j.bpj.2025.12.005.

REFERENCES

1. Keating, C. D. 2012. Aqueous phase separation as a possible route to compartmentalization of biological molecules. *Acc. Chem. Res.* 45:2114–2124.
2. Shin, Y., and C. P. Brangwynne. 2017. Liquid phase condensation in cell physiology and disease. *Science.* 357:eaaf4382.
3. Banani, S. F., H. O. Lee, …, M. K. Rosen. 2017. Biomolecular condensates: organizers of cellular biochemistry. *Nat. Rev. Mol. Cell Biol.* 18:285–298.
4. Dignon, G. L., R. B. Best, and J. Mittal. 2020. Biomolecular phase separation: from molecular driving forces to macroscopic properties. *Annu. Rev. Phys. Chem.* 71:53–75.
5. Jacobs, W. M., and D. Frenkel. 2017. Phase transitions in biological systems with many components. *Biophys. J.* 112:683–691.
6. Elbaum-Garfinkle, S., Y. Kim, …, C. P. Brangwynne. 2015. The disordered P granule protein LAF-1 drives phase separation into droplets with tunable viscosity and dynamics. *P. Natl. Acad. Sci. USA.* 112:7189–7194.

7. Burke, K. A., A. M. Janke, …, N. L. Fawzi. 2015. Residue-by-residue view of in vitro FUS granules that bind the C-terminal domain of RNA polymerase II. *Mol. Cell.* 60:231–241.

8. Forman-Kay, J. D., R. W. Kriwacki, and G. Seydoux. 2018. Phase separation in biology and disease. *J. Mol. Biol.* 430:4603–4606.

9. Wang, H., F. M. Kelley, …, Z. Shi. 2021. Surface tension and viscosity of protein condensates quantified by micropipette aspiration. *Biophys. Rep.* 1:100011.

10. Ambadi Thody, S., H. D. Clements, …, M. K. Rosen. 2024. Small-molecule properties define partitioning into biomolecular condensates. *Nat. Chem.* 16:1794–1802.

11. Chong, P. A., and J. D. Forman-Kay. 2016. Liquid–liquid phase separation in cellular signaling systems. *Curr. Opin. Struct. Biol.* 41:180–186.

12. Garabedian, M. V., W. Wang, …, M. C. Good. 2021. Designer membraneless organelles sequester native factors for control of cell behavior. *Nat. Chem. Biol.* 17:998–1007.

13. Keyport Kik, S., D. Christopher, …, D. A. Drummond. 2024. An adaptive biomolecular condensation response is conserved across environmentally divergent species. *Nat. Commun.* 15:3127.

14. Hoffmann, C., J. Rentsch, …, D. Milovanovic. 2023. Synapsin condensation controls synaptic vesicle sequestering and dynamics. *Nat. Commun.* 14:6730.

15. Cai, D., Z. Liu, and J. Lippincott-Schwartz. 2021. Biomolecular condensates and their links to cancer progression. *Trends Biochem. Sci.* 46:535–549.

16. Zhang, H., S. Elbaum-Garfinkle, …, A. S. Gladfelter. 2015. RNA controls PolyQ protein phase transitions. *Mol. Cell.* 60:220–230.

17. Lin, Y., D. S. W. Protter, …, R. Parker. 2015. Formation and maturation of phase-separated liquid droplets by RNA-binding proteins. *Mol. Cell.* 60:208–219.

18. Patel, A., H. O. Lee, …, S. Alberti. 2015. A liquid-to-solid phase transition of the ALS protein FUS accelerated by disease mutation. *Cell.* 162:1066–1077.

19. Banani, S. F., A. M. Rice, …, M. K. Rosen. 2016. Compositional control of phase-separated cellular bodies. *Cell.* 166:651–663.

20. Frey, S., R. Rees, …, D. Görlich. 2018. Surface properties determining passage rates of proteins through nuclear pores. *Cell.* 174:202–217.e9.

21. Xing, W., D. Muhlrad, …, M. K. Rosen. 2020. A quantitative inventory of yeast P body proteins reveals principles of composition and specificity. *eLife.* 9:e56525.

22. Kelley, F. M., A. Ani, …, B. S. Schuster. 2025. Controlled and orthogonal partitioning of large particles into biomolecular condensates. *Nat. Commun.* 16:3521.

23. McCall, P. M., K. Kim, …, J. Brugués. 2025. A label-free method for measuring the composition of multicomponent biomolecular condensates. *Nat. Chem.* 17:1891–1902.

24. Qian, D., H. Ausserwoger, …, T. P. J. Knowles. 2024. Dominance analysis to assess solute contributions to multicomponent phase equilibria. *P. Natl. Acad. Sci. USA.* 121:e2407453121.

25. Mukherjee, S., and L. V. Schäfer. 2023. Thermodynamic forces from protein and water govern condensate formation of an intrinsically disordered protein domain. *Nat. Commun.* 14:5892.

26. Mukherjee, S., S. Ramos, …, L. V. Schäfer. 2024. Entropy tug-of-war determines solvent effects in the liquid–liquid phase separation of a globular protein. *J. Phys. Chem. Lett.* 15:4047–4055.

27. Pezzotti, S., B. König, …, M. Havenith. 2023. Liquid–liquid phase separation? Ask the water. *J. Phys. Chem. Lett.* 14:1556–1563.

28. Lin, Y. H., J. P. Brady, …, H. S. Chan. 2017. Charge pattern matching as a 'fuzzy' mode of molecular recognition for the functional phase separations of intrinsically disordered proteins. *New J. Phys.* 19:115003.

29. Wang, J., J.-M. Choi, …, A. A. Hyman. 2018. A molecular grammar governing the driving forces for phase separation of prion-like RNA binding proteins. *Cell.* 174:688–699.e16.

30. Dignon, G. L., W. Zheng, …, J. Mittal. 2018. Sequence determinants of protein phase behavior from a coarse-grained model. *PLoS Comput. Biol.* 14:e1005941.

31. Rekhi, S., C. G. Garcia, …, J. Mittal. 2024. Expanding the molecular language of protein liquid–liquid phase separation. *Nat. Chem.* 16:1113–1124.

32. Murthy, A. C., G. L. Dignon, …, N. L. Fawzi. 2019. Molecular interactions underlying liquid- liquid phase separation of the FUS low-complexity domain. *Nat. Struct. Mol. Biol.* 26:637–648.

33. Quiroz, F. G., and A. Chilkoti. 2015. Sequence heuristics to encode phase behaviour in intrinsically disordered protein polymers. *Nat. Mater.* 14:1164–1171.

34. Gu, C., A. Vovk, …, A. Zilman. 2019. The role of cohesiveness in the permeability of the spatial assemblies of FG nucleoporins. *Biophys. J.* 116:1204–1215.

35. Riback, J. A., M. A. Bowman, …, T. R. Sosnick. 2017. Innovative scattering analysis shows that hydrophobic disordered proteins are expanded in water. *Science.* 358:238–241.

36. Yeong, V., J.-W. Wang, …, A. C. Obermeyer. 2022. Intracellular phase separation of globular proteins facilitated by short cationic peptides. *Nat. Commun.* 13:7882.

37. Lau, H. K., L. Li, …, K. L. Kiick. 2017. Aqueous liquid–liquid phase separation of resilin-like polypeptide/polyethylene glycol solutions for the formation of microstructured hydrogels. *ACS Biomater. Sci. Eng.* 3:757–766.

38. Ahn, S. Y., and A. C. Obermeyer. 2024. Selectivity of Complex Coacervation in Multiprotein Mixtures. *JACS Au.* 4:3800–3812.

39. Feric, M., N. Vaidya, …, C. P. Brangwynne. 2016. Coexisting liquid phases underlie nucleolar subcompartments. *Cell.* 165:1686–1697.

40. Abramson, J., J. Adler, …, J. M. Jumper. 2024. Accurate structure prediction of biomolecular interactions with AlphaFold 3. *Nature.* 630:493–500.

41. Lazaridis, T., and M. Karplus. 1999. Effective energy function for proteins in solution. *Proteins.* 35:133–152.

42. Jurrus, E., D. Engel, …, N. A. Baker. 2018. Improvements to the APBS biomolecular solvation software suite. *Protein Sci.* 27:112–128.

43. Héctor Sánchez-Morán, J. S., D. K. S. Weltz, and J. L. Kaar. 2021. Understanding Design Rules for Optimizing the Interface between Immobilized Enzymes and Random Copolymer Brushes. *ACS Appl. Mater. Interfaces.* 13:26694–26703.

44. Rego, N. B., E. Xi, and A. J. Patel. 2021. Identifying hydrophobic protein patches to inform protein interaction interfaces. *P. Natl. Acad. Sci. USA.* 118:e2018234118.

45. Rego, N. B., A. L. Ferguson, and A. J. Patel. 2022. Learning the relationship between nanoscale chemical patterning and hydrophobicity. *P. Natl. Acad. Sci. USA.* 119:e2200018119.

46. Acharya, H., S. Vembanur, …, S. Garde. 2010. Mapping hydrophobicity at the nanoscale: Applications to heterogeneous surfaces and proteins. *Faraday Discuss.* 146:353–401.

47. Eastman, P., R. Galvelis, …, T. E. Markland. 2023. OpenMM 8: molecular dynamics simulation with machine learning potentials. *J. Phys. Chem. B.* 128:109–116.

48. Maier, J. A., C. Martinez, …, C. Simmerling. 2015. ff14SB: improving the accuracy of protein side chain and backbone parameters from ff99SB. *J. Chem. Theor. Comput.* 11:3696–3713.

49. Mongan, J., C. Simmerling, …, A. Onufriev. 2007. Generalized Born model with a simple, robust molecular volume correction. *J. Chem. Theor. Comput.* 3:156–169.

50. Urry, D. W., D. C. Gowda, …, R. D. Harris. 1992. Hydrophobicity scale for proteins based on inverse temperature transitions. *Biopolymers.* 32:1243–1250.

51. Kyte, J., and R. F. Doolittle. 1982. A simple method for displaying the hydropathic character of a protein. *J. Mol. Biol.* 157:105–132.

52. Kapcha, L. H., and P. J. Rossky. 2014. A simple atomic-level hydrophobicity scale reveals protein interfacial structure. *J. Mol. Biol.* 426:484–498.

53. Regy, R. M., J. Thompson, …, J. Mittal. 2021. Improved coarse-grained model for studying sequence dependent phase separation of disordered proteins. *Protein Sci.* 30:1371–1379.

54. Tesei, G., T. K. Schulze, …, K. Lindorff-Larsen. 2021. Accurate model of liquid–liquid phase behavior of intrinsically disordered proteins from optimization of single-chain properties. *P. Natl. Acad. Sci. USA.* 118:e2111696118.

55. Villegas, J. A., and E. D. Levy. 2022. A unified statistical potential reveals that amino acid stickiness governs nonspecific recruitment of client proteins into condensates. *Protein Sci.* 31:e4361.

56. Remsing, R. C., E. Xi, and A. J. Patel. 2018. Protein hydration thermodynamics: The influence of flexibility and salt on hydrophobin II hydration. *J. Phys. Chem. B.* 122:3635–3646.

57. Fennell, C. J., C. Kehoe, and K. A. Dill. 2010. Oil/water transfer is partly driven by molecular shape, not just size. *J. Am. Chem. Soc.* 132:234–240.

58. Riback, J. A., L. Zhu, …, C. P. Brangwynne. 2020. Composition-dependent thermodynamics of intracellular phase separation. *Nature.* 581:209–214.

59. De Sancho, D., and X. López. 2025. Crossover in Aromatic Amino Acid Interaction Strength: Tyrosine vs. Phenylalanine in Biomolecular Condensates. *eLife.* 14:RP104950.

60. Auton, M., and D. W. Bolen. 2007. Application of the transfer model to understand how naturally occurring osmolytes affect protein stability. *Methods Enzymol.* 428:397–418.

61. Martin, E. W., A. S. Holehouse, …, T. Mittag. 2020. Valence and patterning of aromatic residues determine the phase behavior of prion-like domains. *Science.* 367:694–699.

62. Kilgore, H. R., P. G. Mikhael, …, R. A. Young. 2024. Distinct chemical environments in biomolecular condensates. *Nat. Chem. Biol.* 20:291–301.

63. Brooks, B. R., C. L. Brooks, III, …, M. Karplus. 2009. CHARMM: the biomolecular simulation program. *J. Comput. Chem.* 30:1545–1614.

64. Nguyen, H., D. R. Roe, and C. Simmerling. 2013. Improved generalized born solvent model parameters for protein simulations. *J. Chem. Theor. Comput.* 9:2020–2034.

65. Steinbrecher, T., and A. Labahn. 2010. Towards accurate free energy calculations in ligand protein-binding studies. *Curr. Med. Chem.* 17:767–785.

66. Srivastava, R., M. Chattopadhyaya, and P. Bandyopadhyay. 2020. Calculation of salt-dependent free energy of binding of β-lactoglobulin homodimer formation and mechanism of dimer formation using molecular dynamics simulation and three-dimensional reference interaction site model (3D-RISM): diffuse salt ions and non-polar interactions between the monomers favor the dimer formation. *Phys. Chem. Chem. Phys.* 22:2142–2156.

67. Rekhi, S., and J. Mittal. 2025. Amino acid transfer free energies reveal thermodynamic driving forces in biomolecular condensate formation. *P. Natl. Acad. Sci. USA.* 122:e2425422122.

68. Schuster, B. S., G. L. Dignon, …, J. Mittal. 2020. Identifying sequence perturbations to an intrinsically disordered protein that determine its phase-separation behavior. *P. Natl. Acad. Sci. USA.* 117:11421–11431.

69. Rubinstein, M., and A. V. Dobrynin. 1997. Solutions of associative polymers. *Trends Polym. Sci.* 5:181–186.

70. Choi, J.-M., A. S. Holehouse, and R. V. Pappu. 2020. Physical principles underlying the complex biology of intracellular phase transitions. *Annu. Rev. Biophys.* 49:107–133.

71. Vernon, R. M., P. A. Chong, …, J. D. Forman-Kay. 2018. Pi-Pi contacts are an overlooked protein feature relevant to phase separation. *eLife.* 7:e31486.

72. Das, S., Y.-H. Lin, …, H. S. Chan. 2020. Comparative roles of charge, π, and hydrophobic interactions in sequence-dependent phase separation of intrinsically disordered proteins. *P. Natl. Acad. Sci. USA.* 117:28795–28805.

73. Joseph, J. A., A. Reinhardt, …, R. Collepardo-Guevara. 2021. Physics-driven coarse-grained model for biomolecular phase separation with near-quantitative accuracy. *Nat. Comput. Sci.* 1:732–743.

Biophysical Journal, Volume *125*

Supplemental information

Hydration free energy is an incomplete predictor of globular protein incorporation into condensates

Sophie Anderson, Malcolm Harrison, and Gregory L. Dignon

Supporting Information: Hydration free energy is an incomplete predictor of globular protein incorporation into condensates

Sophie Anderson[1,2], Malcolm Harrison[3], and Gregory L Dignon[3,*]

[1]Department of Biochemistry, Vassar College, Poughkeepsie, NY 12604, United States
[2]Current Address: Department of Epidemiology Columbia University, New York, NY 10032, United States.
[3]Department of Chemical and Biochemical Engineering, Rutgers University, Piscataway, NJ 08854, United States
[*]Correspondence: gregory.dignon@rutgers.edu

1. Supplementary Figures

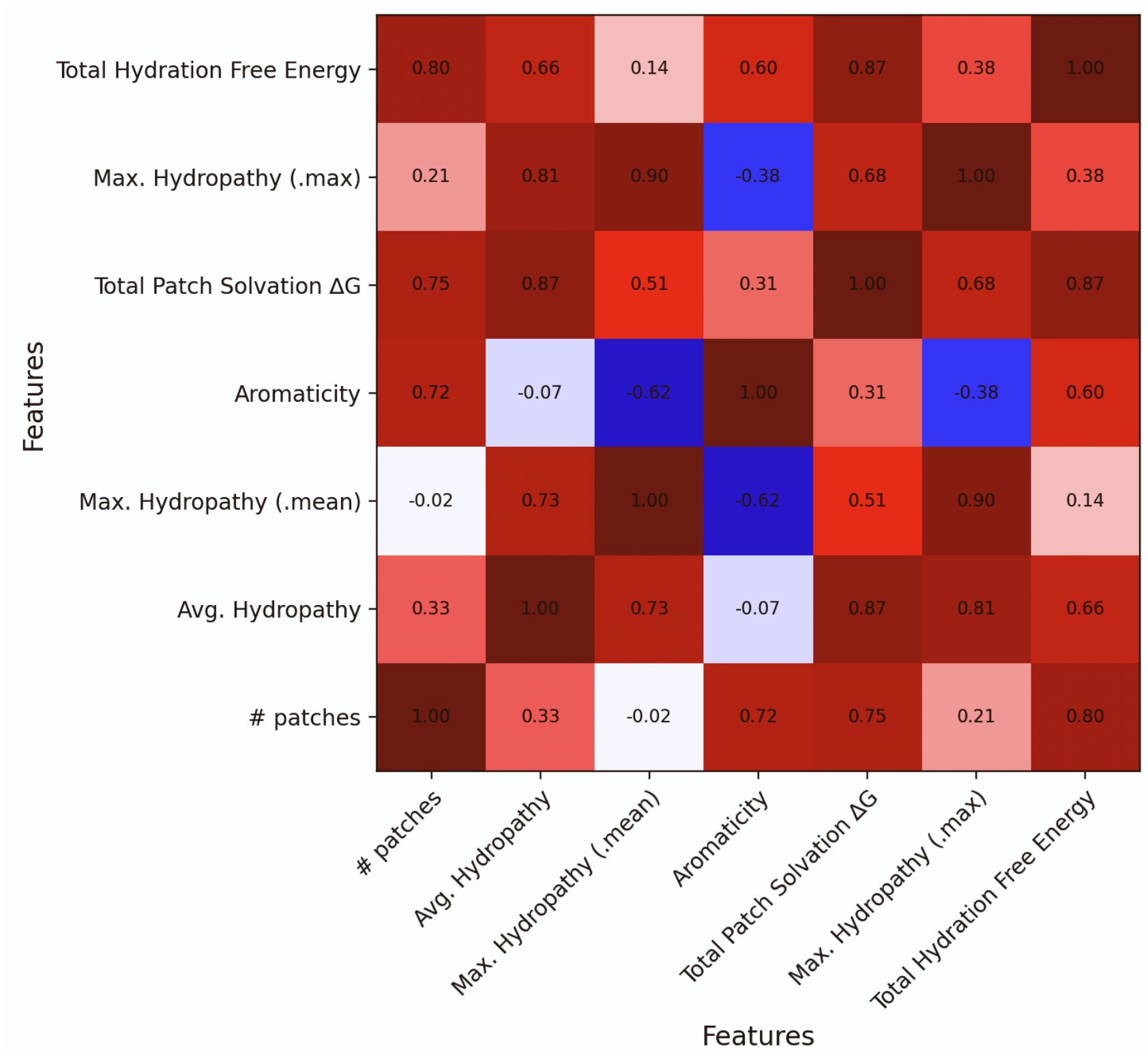

Fig S1: Analysis of different outputs from hi-patch software[3] (See Table S1) and their relatedness when applied to the 17 variants of GFP. Covariance will be 1 if parameters are perfectly correlated, and -1 if perfectly anticorrelated. A value close to 0 indicates they are uncorrelated.

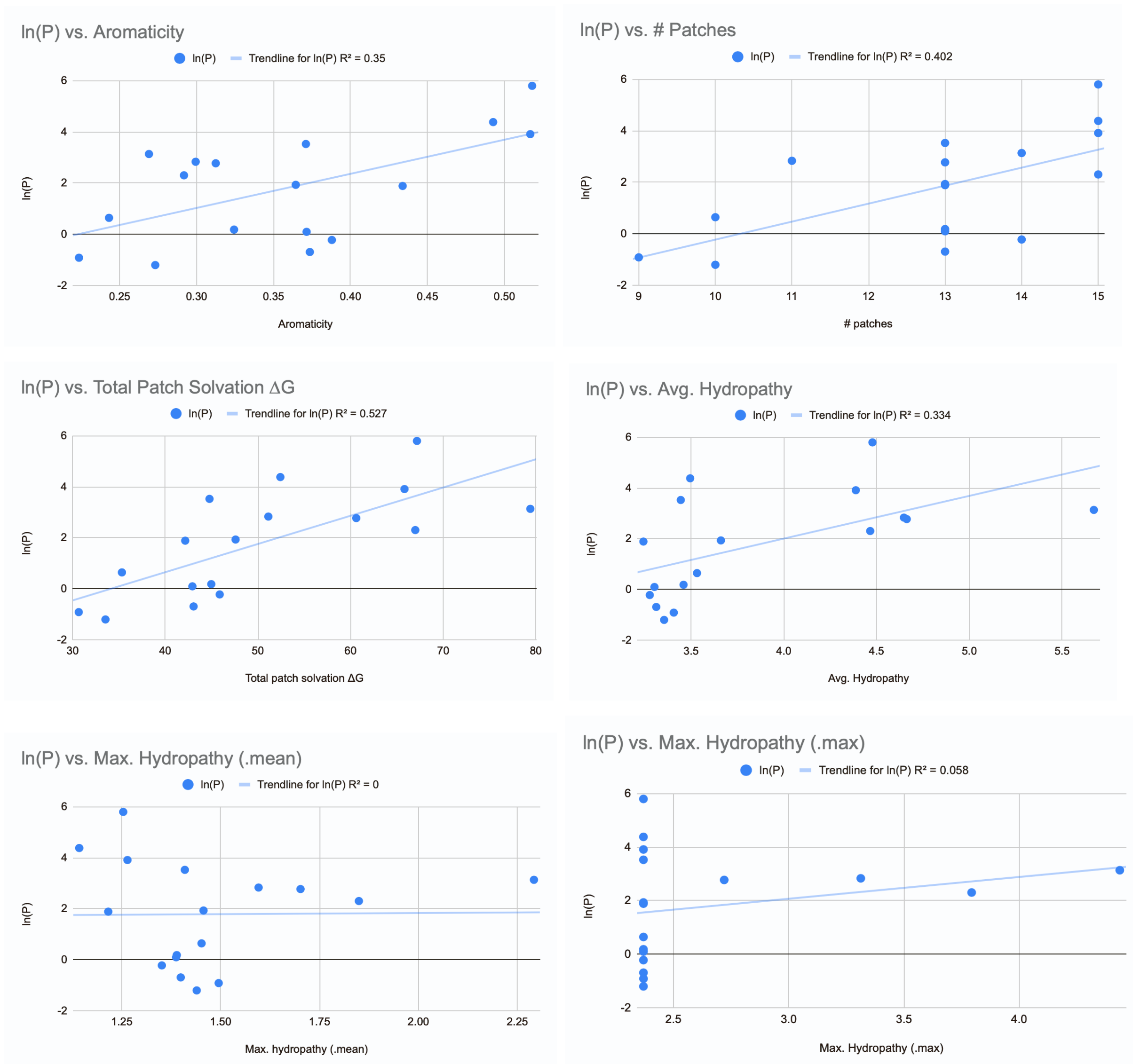

Fig. S2: predictions of ln(P) from other hi-patch outputs are not as robust as with the full hydration free energy calculation.

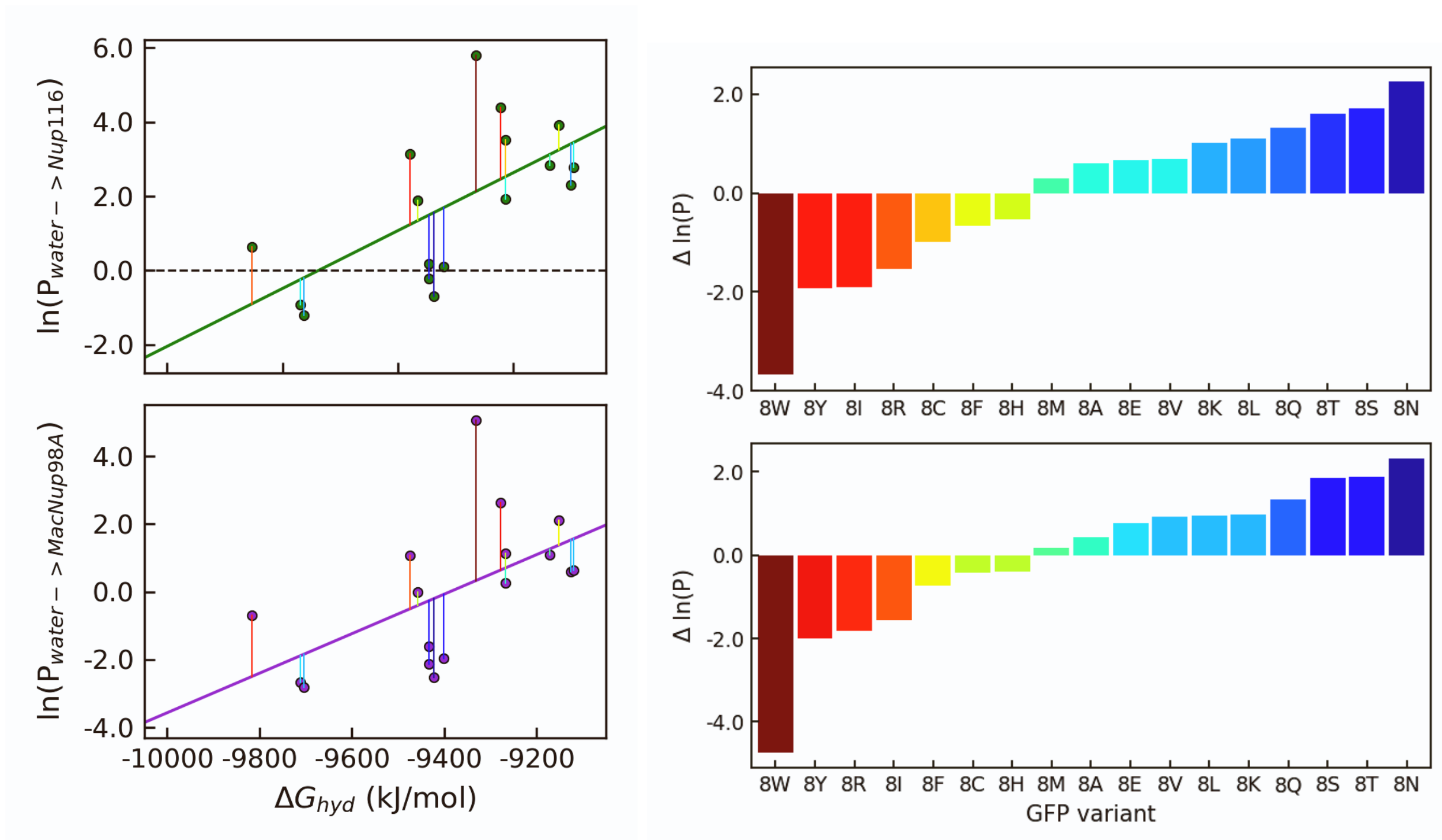

Fig. S3: Predictive model and residuals for static calculation of hydration free energy using the eef1 force field implemented in CHARMM[4].

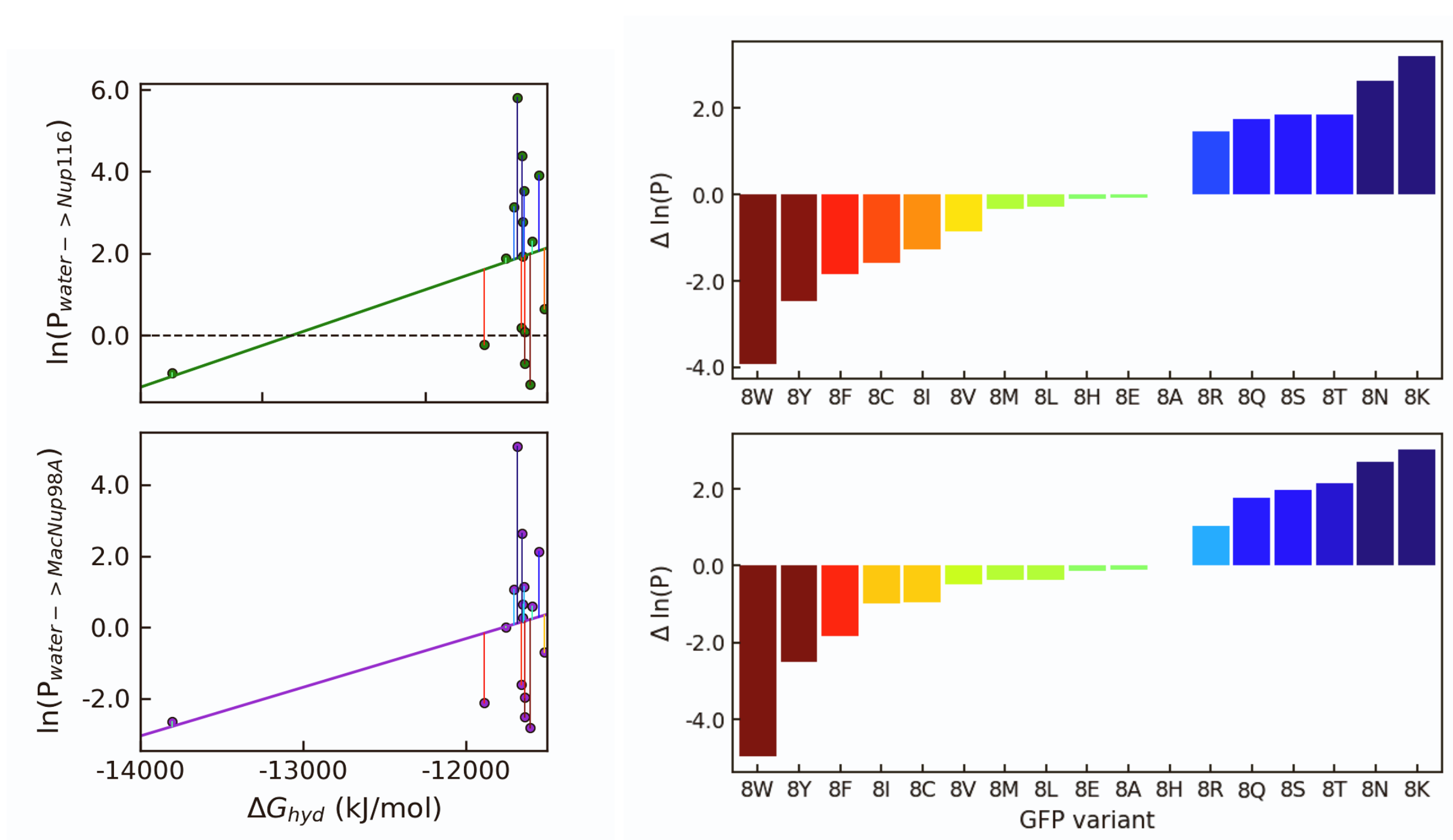

Fig S4: Predictive model and residuals for static calculation of hydration free energy using the adaptive Poisson-Boltzmann solver (APBS)[5].

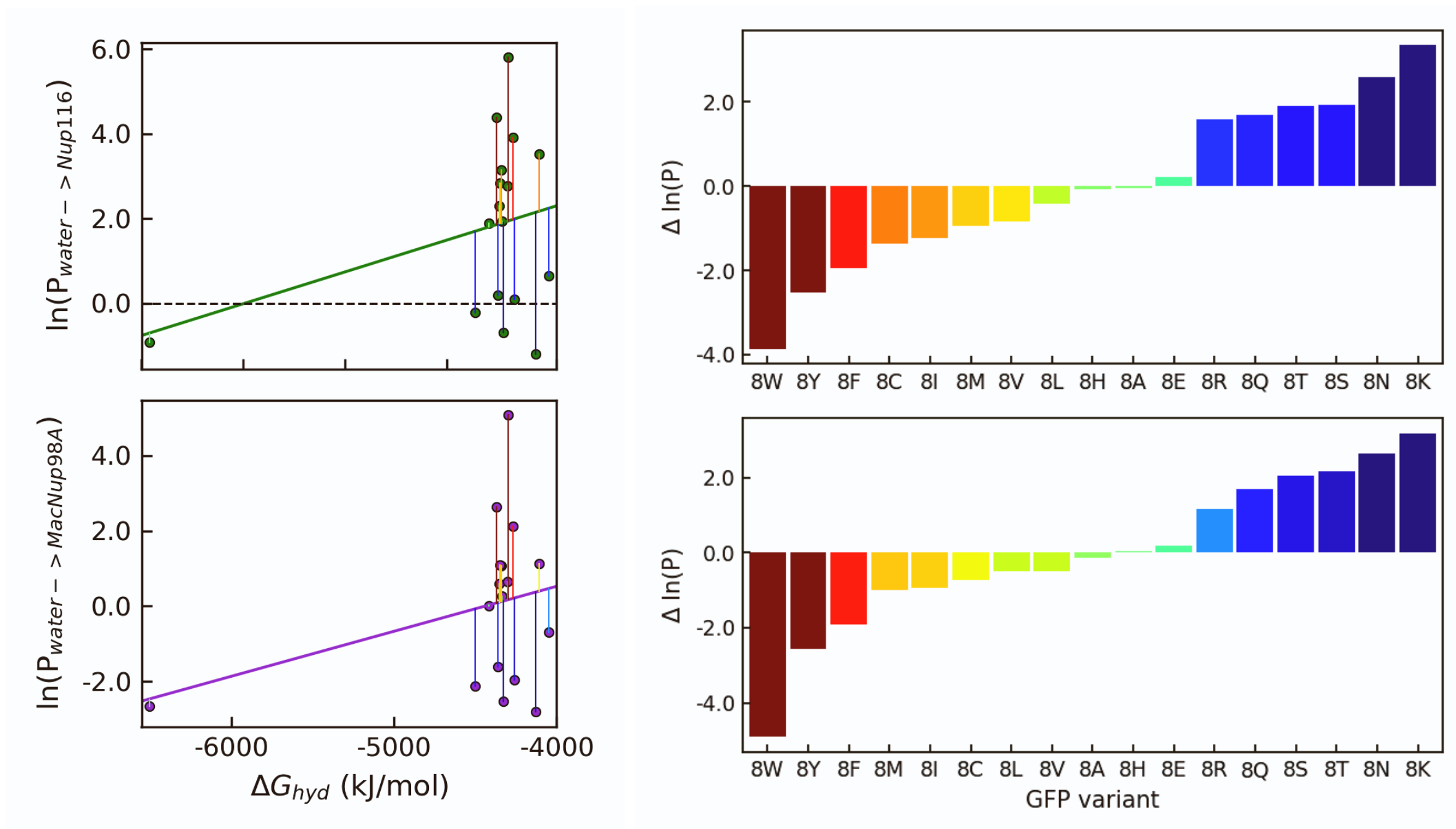

Fig. S5: Predictive model and residuals for dynamic hydration free energy calculations using MD simulation with GBNeck implicit solvent[6].

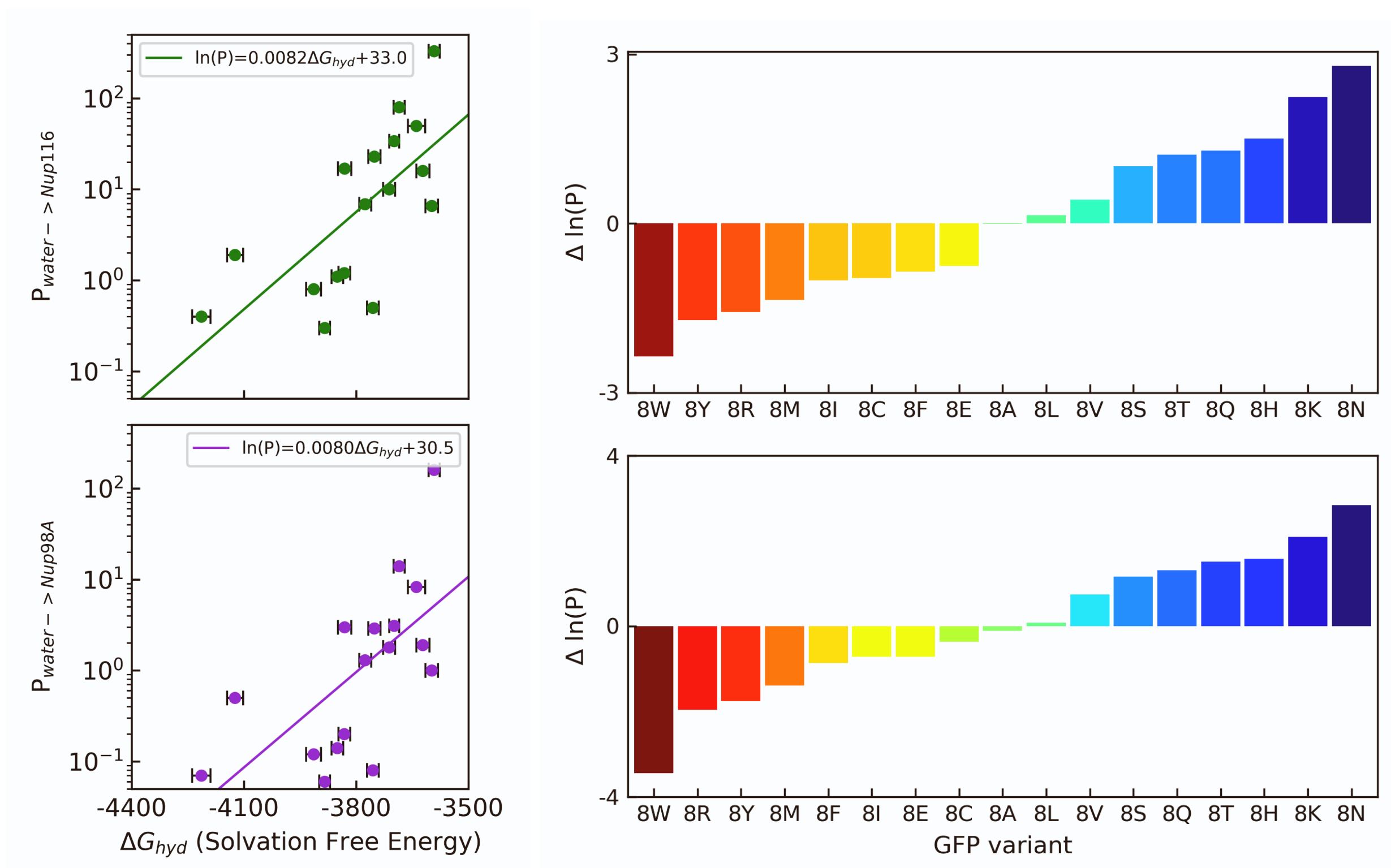

Fig. S6: Predictive model and residuals for dynamic explicit solvent simulation trajectory analyzed using hi-patch to calculate hydration free energy. Error bars are SEM from the simulation calculated by block averaging with 5 blocks.

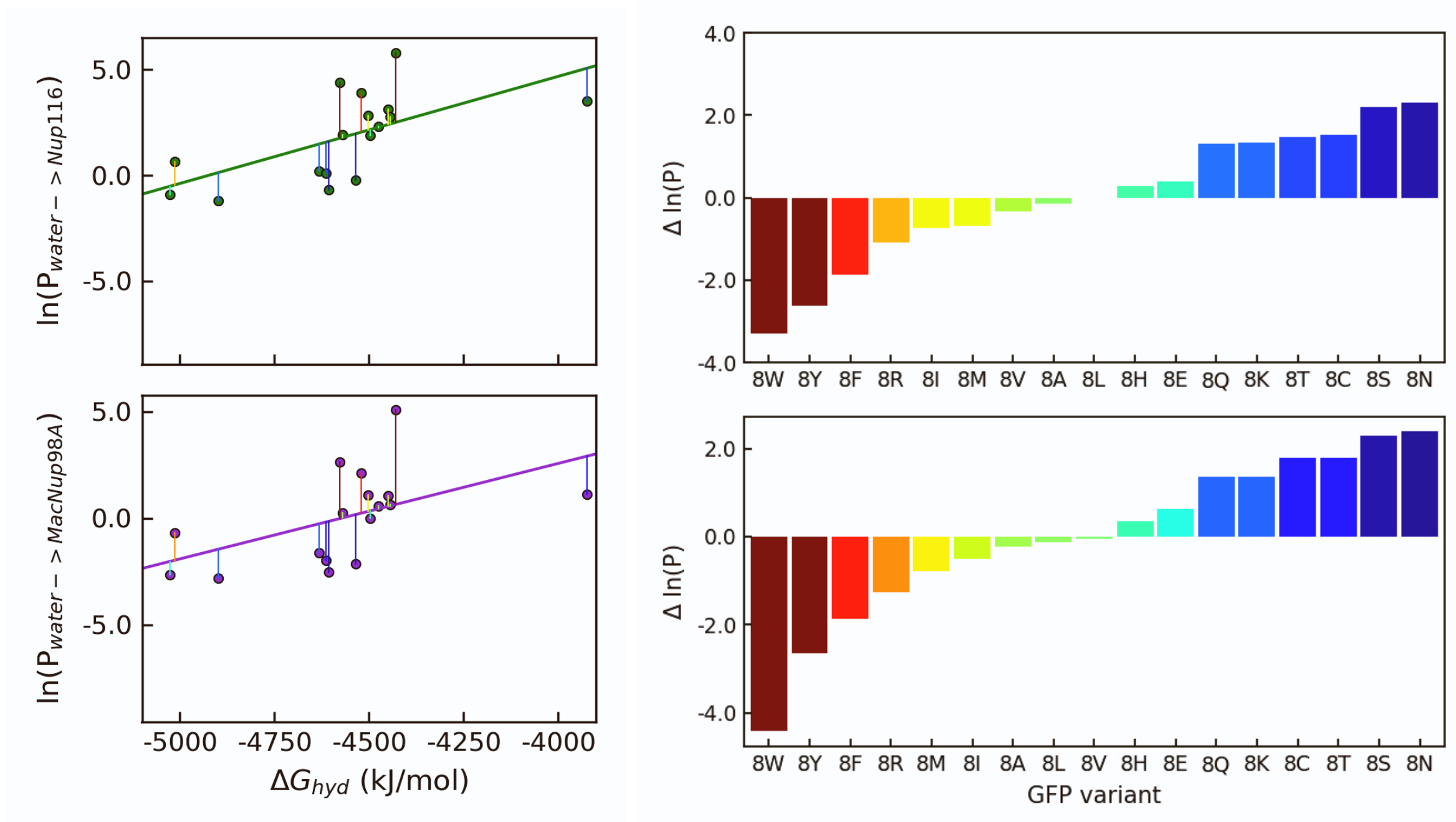

Figure S7: Predictive model and residuals for dynamic implicit solvent simulation trajectory analyzed by hi-patch to calculate hydration free energy.

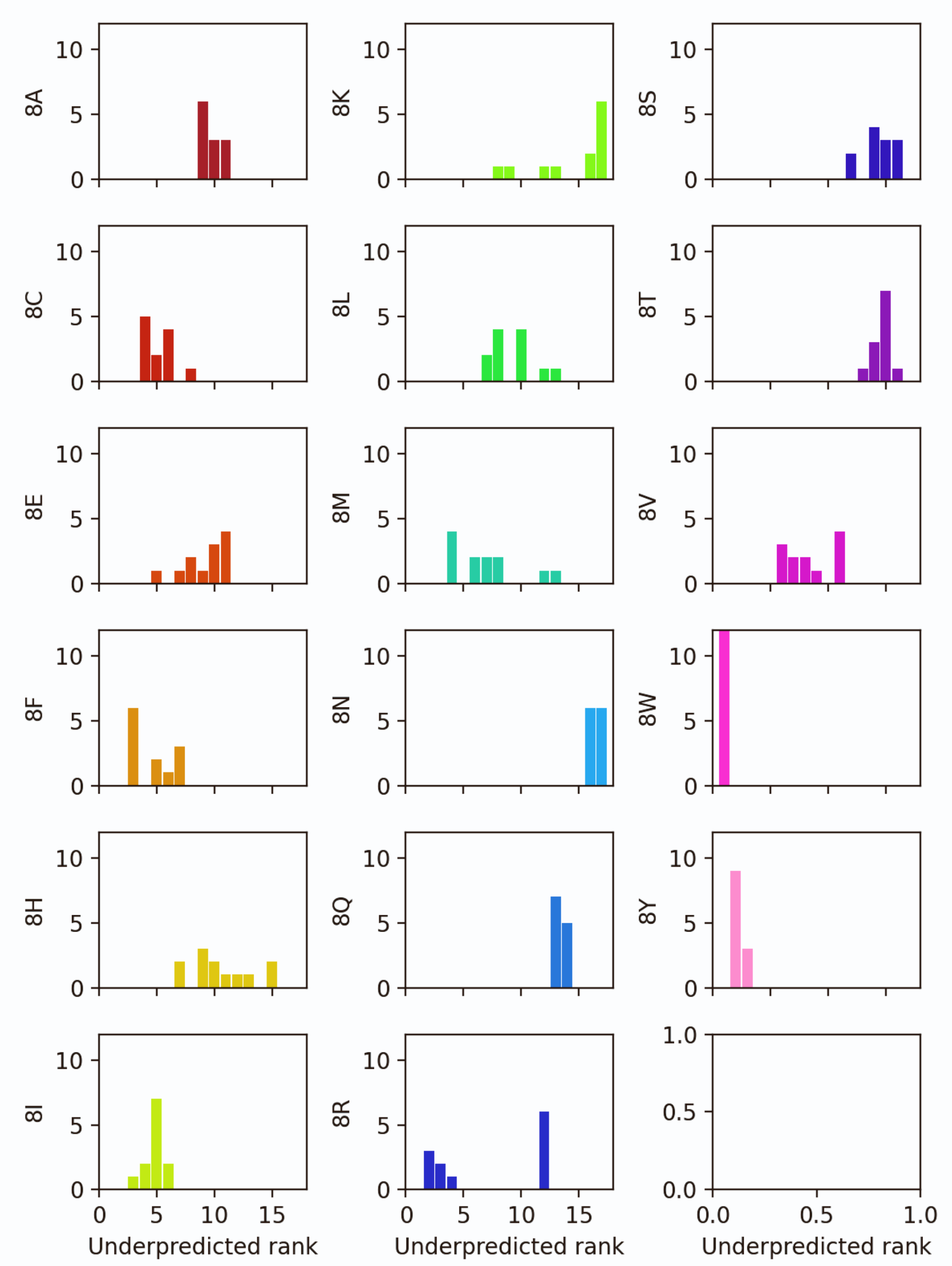

Fig. S8: For all six hydration free energy methods tested, we calculated the ranking of overpredicted and underpredicted proteins and show the histogram of ranks for each GFP variant. Having density toward low numbers indicates underpredictedness, and greater specific interactions between the residue type and scaffold proteins

2. Supporting Tables

Table S1: List of GFP variants from Frey et al.[1] and their amino acid sequences used in modeling of the globular proteins in this work.

Variant	Sequence
**efGFP**	GEELFTGVVPILVELDGDVNGHKFSVRGEGEGDATYGKLTLKFICTTGKLPVPWPTLV<br>TTLTYGVQCFSHYPDHMKQHDFFKSAMPEGYVQERTIYFEDDGAYKTRAEVKFEGDTL<br>VNRIELKGIDFKEDGNILGHKLEYNFNSHNVYIMADKQKNGIKANFNTRHNVEDGSEQ<br>VADHYQQNTPIGDGPVLLPDNHYLNTQSKLSKDPNEKRDHMVLKEFVTAAGI
**8A**	GEELFTGVVPILVELDGDVNGHKFSVRGEGEGDATYGALTLKFICTTGKLPVPWPTLV<br>TTLTYGVQCFSHYPDHMKQHDFFKSAMPEGYVQERAIYFEDDGAYKTRAEVKFEGDTL<br>VNRIELKGIDFKEDGNILGHKLEYNFNSHAVYIMADKQKNGIKAAFNTRHNVEDGSEQ<br>VAAHYQQNTPIGDGPVLLPDNHYLNTASKLSADPNEKRDHMVLKEFVAAAGI
**8C**	GEELFTGVVPILVELDGDVNGHKFSVRGEGEGDATYGCLTLKFICTTGKLPVPWPTLV<br>TTLTYGVQCFSHYPDHMKQHDFFKSAMPEGYVQERCIYFEDDGAYKTRAEVKFEGDTL<br>VNRIELKGIDFKEDGNILGHKLEYNFNSHCVYIMADKQKNGIKACFNTRHNVEDGSEQ<br>VACHYQQNTPIGDGPVLLPDNHYLNTCSKLSCDPNEKRDHMVLKEFVCAAGI
**8E**	GEELFTGVVPILVELDGDVNGHKFSVRGEGEGDATYGELTLKFICTTGKLPVPWPTLV<br>TTLTYGVQCFSHYPDHMKQHDFFKSAMPEGYVQEREIYFEDDGAYKTRAEVKFEGDTL<br>VNRIELKGIDFKEDGNILGHKLEYNFNSHEVYIMADKQKNGIKAEFNTRHNVEDGSEQ<br>VAEHYQQNTPIGDGPVLLPDNHYLNTESKLSEDPNEKRDHMVLKEFVEAAGI
**8F**	GEELFTGVVPILVELDGDVNGHKFSVRGEGEGDATYGFLTLKFICTTGKLPVPWPTLV<br>TTLTYGVQCFSHYPDHMKQHDFFKSAMPEGYVQERFIYFEDDGAYKTRAEVKFEGDTL<br>VNRIELKGIDFKEDGNILGHKLEYNFNSHFVYIMADKQKNGIKAFFNTRHNVEDGSEQ<br>VAFHYQQNTPIGDGPVLLPDNHYLNTFSKLSFDPNEKRDHMVLKEFVFAAGI
**8H**	GEELFTGVVPILVELDGDVNGHKFSVRGEGEGDATYGHLTLKFICTTGKLPVPWPTLV<br>TTLTYGVQCFSHYPDHMKQHDFFKSAMPEGYVQERHIYFEDDGAYKTRAEVKFEGDTL<br>VNRIELKGIDFKEDGNILGHKLEYNFNSHHVYIMADKQKNGIKAHFNTRHNVEDGSEQ<br>VAHHYQQNTPIGDGPVLLPDNHYLNTHSKLSHDPNEKRDHMVLKEFVHAAGI
**8I**	GEELFTGVVPILVELDGDVNGHKFSVRGEGEGDATYGILTLKFICTTGKLPVPWPTLV<br>TTLTYGVQCFSHYPDHMKQHDFFKSAMPEGYVQERIIYFEDDGAYKTRAEVKFEGDTL<br>VNRIELKGIDFKEDGNILGHKLEYNFNSHIVYIMADKQKNGIKAIFNTRHNVEDGSEQ<br>VAIHYQQNTPIGDGPVLLPDNHYLNTISKLSIDPNEKRDHMVLKEFVIAAGI
**8K**	GEELFTGVVPILVELDGDVNGHKFSVRGEGEGDATYGHLTLKFICTTGKLPVPWPTLV<br>TTLTYGVQCFSHYPDHMKQHDFFKSAMPEGYVQERKIYFEDDGAYKTRAEVKFEGDTL<br>VNRIELKGIDFKEDGNILGHKLEYNFNSHKVYIMADKQKNGIKAKFNTRHNVEDGSEQ<br>VAKHYQQNTPIGDGPVLLPDNHYLNTKSKLSKDPNEKRDHMVLKEFVKAAGI

**8L**	GEELFTGVVPILVELDGDVNGHKFSVRGEGEGDATYGLLTLKFICTTGKLPVPWPTLV<br>TTLTYGVQCFSHYPDHMKQHDFFKSAMPEGYVQERLIYFEDDGAYKTRAEVKFEGDTL<br>VNRIELKGIDFKEDGNILGHKLEYNFNSHLVYIMADKQKNGIKALFNTRHNVEDGSEQ<br>VALHYQQNTPIGDGPVLLPDNHYLNTLSKLSLDPNEKRDHMVLKEFVLAAGI
**8M**	GEELFTGVVPILVELDGDVNGHKFSVRGEGEGDATYGMLTLKFICTTGKLPVPWPTLV<br>TTLTYGVQCFSHYPDHMKQHDFFKSAMPEGYVQERMIYFEDDGAYKTRAEVKFEGDTL<br>VNRIELKGIDFKEDGNILGHKLEYNFNSHMVYIMADKQKNGIKAMFNTRHNVEDGSEQ<br>VAMHYQQNTPIGDGPVLLPDNHYLNTMSKLSMDPNEKRDHMVLKEFVMAAGI
**8N**	GEELFTGVVPILVELDGDVNGHKFSVRGEGEGDATYGNLTLKFICTTGKLPVPWPTLV<br>TTLTYGVQCFSHYPDHMKQHDFFKSAMPEGYVQERNIYFEDDGAYKTRAEVKFEGDTL<br>VNRIELKGIDFKEDGNILGHKLEYNFNSHNVYIMADKQKNGIKANFNTRHNVEDGSEQ<br>VANHYQQNTPIGDGPVLLPDNHYLNTNSKLSNDPNEKRDHMVLKEFVNAAGI
**8Q**	GEELFTGVVPILVELDGDVNGHKFSVRGEGEGDATYGQLTLKFICTTGKLPVPWPTLV<br>TTLTYGVQCFSHYPDHMKQHDFFKSAMPEGYVQERQIYFEDDGAYKTRAEVKFEGDTL<br>VNRIELKGIDFKEDGNILGHKLEYNFNSHQVYIMADKQKNGIKAQFNTRHNVEDGSEQ<br>VAQHYQQNTPIGDGPVLLPDNHYLNTQSKLSQDPNEKRDHMVLKEFVQAAGI
**8R**	GEELFTGVVPILVELDGDVNGHKFSVRGEGEGDATYGRLTLKFICTTGKLPVPWPTLV<br>TTLTYGVQCFSHYPDHMKQHDFFKSAMPEGYVQERRIYFEDDGAYKTRAEVKFEGDTL<br>VNRIELKGIDFKEDGNILGHKLEYNFNSHRVYIMADKQKNGIKARFNTRHNVEDGSEQ<br>VARHYQQNTPIGDGPVLLPDNHYLNTRSKLSRDPNEKRDHMVLKEFVRAAGI
**8S**	GEELFTGVVPILVELDGDVNGHKFSVRGEGEGDATYGSLTLKFICTTGKLPVPWPTLV<br>TTLTYGVQCFSHYPDHMKQHDFFKSAMPEGYVQERSIYFEDDGAYKTRAEVKFEGDTL<br>VNRIELKGIDFKEDGNILGHKLEYNFNSHSVYIMADKQKNGIKASFNTRHNVEDGSEQ<br>VADSYQQNTPIGDGPVLLPDNHYLNTSSKLSSDPNEKRDHMVLKEFVSAAGI
**8T**	GEELFTGVVPILVELDGDVNGHKFSVRGEGEGDATYGKLTLKFICTTGKLPVPWPTLV<br>TTLTYGVQCFSHYPDHMKQHDFFKSAMPEGYVQERTIYFEDDGAYKTRAEVKFEGDTL<br>VNRIELKGIDFKEDGNILGHKLEYNFNSHNVYIMADKQKNGIKATFNTRHNVEDGSEQ<br>VATHYQQNTPIGDGPVLLPDNHYLNTTSKLSTDPNEKRDHMVLKEFVTAAGI
**8V**	GEELFTGVVPILVELDGDVNGHKFSVRGEGEGDATYGVLTLKFICTTGKLPVPWPTLV<br>TTLTYGVQCFSHYPDHMKQHDFFKSAMPEGYVQERVIYFEDDGAYKTRAEVKFEGDTL<br>VNRIELKGIDFKEDGNILGHKLEYNFNSHVVYIMADKQKNGIKAVFNTRHNVEDGSEQ<br>VAVHYQQNTPIGDGPVLLPDNHYLNTVSKLSVDPNEKRDHMVLKEFVVAAGI
**8W**	GEELFTGVVPILVELDGDVNGHKFSVRGEGEGDATYGWLTLKFICTTGKLPVPWPTLV<br>TTLTYGVQCFSHYPDHMKQHDFFKSAMPEGYVQERWIYFEDDGAYKTRAEVKFEGDTL<br>VNRIELKGIDFKEDGNILGHKLEYNFNSHWVYIMADKQKNGIKAWFNTRHNVEDGSEQ<br>VAWHYQQNTPIGDGPVLLPDNHYLNTWSKLSWDPNEKRDHMVLKEFVWAAGI

8Y	GEELFTGVVPILVELDGDVNGHKFSVRGEGEGDATYGYLTLKFICTTGKLPVPWPTLV TTLTYGVQCFSHYPDHMKQHDFFKSAMPEGYVQERYIYFEDDGAYKTRAEVKFEGDTL VNRIELKGIDFKEDGNILGHKLEYNFNSHYVYIMADKQKNGIKAYFNTRHNVEDGSEQ VAYHYQQNTPIGDGPVLLPDNHYLNTYSKLSYDPNEKRDHMVLKEFVYAAGI

Table S2:Transfer free energies of each GFP into both scaffold proteins calculated from experiments, and hydration free energies from hi-patch. Solvation free energy can be calculated by finding the difference.

**Protein**	**$\Delta F_{tr,\ MacNup98A}$ (kJ/mol)**	**ΔF_{hyd} (kJ/mol)**
8A	-0.7	-3774.2
8C	-2.8	-3810.4
8E	6.6	-4267.8
8F	-5.3	-3660.5
8H	0.0	-3749.6
8I	-2.7	-3796.5
8K	7.0	-4276.2
8L	-1.5	-3777.1
8M	-2.7	-3610.9
8N	6.3	-3870.8
8Q	4.0	-3930.2
8R	1.7	-4200.8
8S	5.3	-3929.4
8T	4.9	-3916.1
8V	-1.6	-3807.2
8W	-12.7	-3616.7
8Y	-6.5	-3723.7

Table S3: List and description of parameters calculated by hi-patch software[3].

# patches	Represents the number of hydrophobic surface regions identified. These patches represent clustered hydrophobic residues exposed to solvent.
Avg. Hydropathy	The mean hydropathy value for each protein variant.
Max. Hydropathy (.mean)	The highest average hydropathy value among all individual surface patches, representing the most hydrophobic surface region for each variant.
Aromaticity	Quantifies the presence of aromatic residues (Tyr, Trp, Phe) on the protein's surface.
Total Patch Solvation ΔG	The total solvation free energy contribution from all identified hydrophobic patches.
Max. Hydropathy (.max)	The single highest hydropathy value at any atomic position on the protein surface, identifying the most hydrophobic individual site for each variant.
Total Hydration Free Energy	The total free energy change (ΔF_{hyd}) when transferring a protein from vacuum to aqueous solution.

Table S4: Sequences for the two Nucleoporin proteins from Frey et al.[1]

**Nucleoporin**	**Sequence**
**MacNup98A**	MFGNTGGGGLFGNTQTQQTGGGLFGQPQQTQFGQTGATGGGLFGGATNTFGGGGG<br>GGLFGGNNNQQTNPTAGGGIFGQGTTGLGGAPAQTGGGLFGAPQNNQGGGLFGGG<br>TTTGGGMFGNQANTQTGGGGLFGGPSQPTTQPPAFSLNNPTTGGGGLFGQPANTM<br>GGNNGGLFGGQTNSFGANNNMLGNNNRPQGAGIFGGATTTAPTGNTGMFGGIGAN<br>NGGGGLFGMNNTNTNPTGGFGATNPTAGGGGLFGGGATTTGGGGLFGGGNTQGGG<br>LLGTANTTAGGLLGGGFNMNNNTGGILGQTNNQFGLGSFGTNNNAAAAPFQPKAS<br>ANGvltkpneknlcyaisngtdfcifelaltqrklvkAGQLKPGAQQAGGMFGQP<br>AQGGNGLFGGGGAATTTPFGGAQNGNLFGGQNTQAQGGGLFGAPVNNAATGAGGG<br>LFGAKPAATTTGGGLFGQMPAQTGGFLGNTATQPAGGGLFGGATTTQAPGGGGGG<br>GLFGGNTTAATTGGGLFGGNTQTGGATGGLFGGQQPNNQGGLFLNTGNANNANTG

	GGLFGGATTTPATGGGLFGGSTNTQPGLATGGGLFGNNQGASQPAAQGGLFGGAA<br>PQQNSLFGGATAGGQTGGLFGGATGATQQQGGGL<br>FGQTASNPTQGGGLFGAANPGLGGAAA
**Nup116**	MFGVSRGAFPSATTQPFGSTGSTFGGQQQQQPVANTSAFGLSQQTNTTQAPAFG<br>NFGNQTSNSPFGMSGSTTANGTPFGQSQLTNNNASGSIFGGMGNNTALSAGSASv<br>vpnstagtsikpfttfeekdpttgvinvfqsitcmpeyrnfsfeelrfqdyqagr<br>kFGTSQNGTGTTFNNPQGTTNTGFGIMGNNNSTTSATTGGLFGQKPATGMFGTGT<br>GSGGGFGSGATNSTGLFGSSTNLSGNSAFGANKPATSGGLFGNTTNNPTNGTNNT<br>GLFGQQNSNTNGGLFGQQQNSFGANNVSNGGAFGQVNRGAFPQQQTQQGSGGIFG<br>QSNANANGGAFGQQQGTGALFGAKPASGGLFGQSAGSKAFGMNTNPTGTTGGLFG<br>QTNQQQSGGGLFGQQQNSNAGGLFGQNNQSQNQSGLFGQQNSSNAFGQPQQQGGL<br>FGSKPAGGLFGQQQGASTFASGNAQNNSIFGQNNQQQQSTGGLFGQQNNQSQSQP<br>GGLFGQTNQNNNQPFGQNGLQQPQQNNSLFGAKPTGFGNTSLFSNSTTNQSNGIS<br>GNNLQQQSGGLFQNKQQPASGGLFGSKPSNTVGGGLFGNNQVANQNNPASTSGGL<br>FGSKPATGSLFGGTNSTAPNASSGGIFGSNNASNTAATTNSTGLFGNKPVGAGAS<br>TSAGGLFGNNNNSSLNNSNGSTGLFGSNNTSQSTNAGGLFQNNTSTNTSGGGLFS<br>QPSQSMAQSQNALQQQQQQR

Table S5: Size, charge and hydropathy[2] of protein sequences used in this work.

Name	Length	Net charge	Urry hydropathy
8A	226	-6	0.573
8C	226	-6	0.575
8E	226	-14	0.552
8F	226	-6	0.581
8H	226	-2	0.579
8I	226	-6	0.577
8K	226	1.5	0.567
8L	226	-6	0.577

8M	226	-6	0.576
8N	226	-6	0.572
8Q	226	-6	0.571
8R	226	2.0	0.571
8S	226	-7.5	0.570
8T	226	-5	0.572
8V	226	-6	0.575
8W	226	-6	0.587
8Y	226	-6	0.583
Nup116	1113	20.5	0.592
MacNup98A	1105	16	0.594

Suporting References

1. Frey, S., R. Rees, J. Schünemann, S. C. Ng, K. Fünfgeld, T. Huyton, and D. Görlich, 2018. Surface properties determining passage rates of proteins through nuclear pores. *Cell* 174:202–217.
2. Urry, D. W., D. C. Gowda, T. M. Parker, C. Luan, M. C. Reid, C. M. Harris, A. Pattanaik, and R. D. Harris, 1992.Hydrophobicity scale for proteins based on inverse temperature transitions. *Biopolymers* 32:1243–1250.
3. Héctor Sánchez-Morán James S. Weltz, D. K. S., and J. L. Kaar, 2021. Understanding Design Rules for Optimizing the Interface between Immobilized Enzymes and Random Copolymer Brushes. *ACS Applied Materials and Interfaces*
4. Lazaridis, T., and M. Karplus, 1999. Effective energy function for proteins in solution. *Proteins: Structure, Function, and Bioinformatics* 35:133–152.
5. Jurrus, E., D. Engel, K. Star, K. Monson, J. Brandi, L. E. Felberg, D. H. Brookes, L. Wilson, J. Chen, K. Liles, et al., 2018. Improvements to the APBS biomolecular solvation software suite. *Protein science* 27:112–128.
6. Mongan, J., C. Simmerling, J. A. McCammon, D. A. Case, and A. Onufriev, 2007. Generalized Born model with a simple, robust molecular volume correction. *Journal of chemical theory and computation* 3:156–169.
